# Supplementary material for: Quantifying the indirect impact of COVID-19 pandemic on utilisation of outpatient and immunisation services in Kenya: a longitudinal study using interrupted time series analysis
Source: BMJ Open. 2022 Mar 10;12(3):e055815. doi: 10.1136/bmjopen-2021-055815 (PMC8914407; doi:10.1136/bmjopen-2021-055815)
Supplement: Supplementary data [file bmjopen-2021-055815supp001.pdf]

SI Figure 1: Missing data patterns plot for indicators showing number of reported months by health facilities.

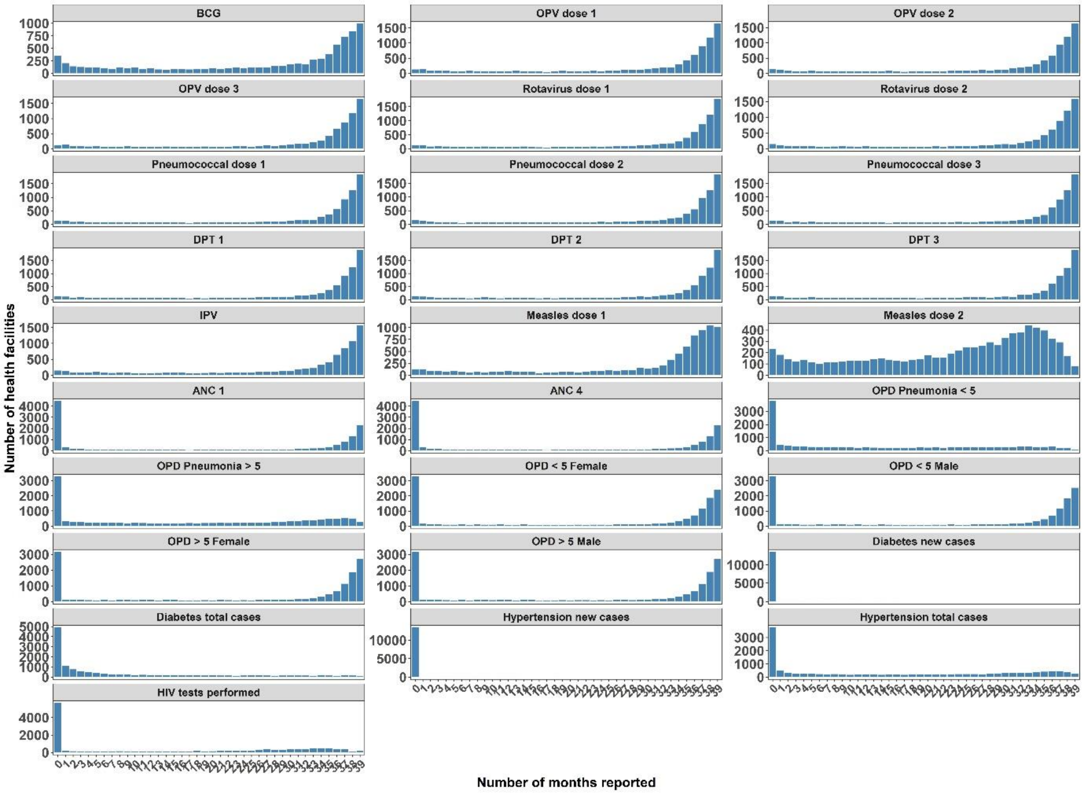

The x – axis shows the number of months reported by health facilities (0 to 39). 0 to the left means the health facilities did not report any month or may not be offering the service, while 39 means the health facilities reported all months.
