# Supplementary material for: Quantifying the indirect impact of COVID-19 pandemic on utilisation of outpatient and immunisation services in Kenya: a longitudinal study using interrupted time series analysis
Source: BMJ Open. 2022 Mar 10;12(3):e055815. doi: 10.1136/bmjopen-2021-055815 (PMC8914407; doi:10.1136/bmjopen-2021-055815)
Supplement: Supplementary data [file bmjopen-2021-055815supp002.pdf]

Additional Information 4

Handling missing data

Missing data occurrence

Missing data occurred for the indicators in a given month for a given health facility.

Health facility ownership (public or private), level of health facility, time (month and year) and COVID-19 binary indicator (0 – months before pandemic and 1 – months post pandemic) were used as covariates (independent variables) with the health facility as a clustering variable. These hospital characteristics were fully observed across all hospitals.

Multiple Imputation under joint modelling framework

We implemented MI under the joint modelling imputation framework. In instances with complete data, standard statistical approaches for multi-level data apply models accounting for this dependency (1). Similarly, for missing values, imputation techniques need to account for dependency between observations, otherwise the predictive variance of the missing data is not accurately reflected. Certainly, if an incomplete variable is imputed ignoring the multilevel structure, the resultant imputations can be unreliable (1), and consequently bias in estimates obtained from imputed data. Therefore, to account for the multilevel structure, imputation techniques based on regression models that include a random intercept for clusters are generally used and have been implemented in the R-package *jomo* (2-4).

General model specification under joint modelling framework

Since missing data was observed in each indicator (outcome variable) in our dataset, with hospital characteristics fully observed (covariates), this presents a univariate missing data pattern (1). The general imputation model under this scenario is outlined below (1);

Let the matrix $Y_{n\times p} = (y_1, \dots., y_p)$ be the matrix of incomplete data for n items in rows and p variables in columns. Let i be the index for the individuals (($1 \le i \le n$ and j for columns ($1 \le j \le p$). Y is stratified to K clusters of size n_k where k denotes the index for a cluster ($1 \le k \le K$). So, y_{jk} denotes the n_k- vector corresponding to vector y_j restricted to individuals within

cluster k. Then, let (y_j^{obs}, y_j^{miss}) be the missing and observed parts of y_j and let $Y^{obs} = (y_1^{obs}, \dots, y_p^{obs})$ and $Y^{miss} = (y_1^{miss}, \dots, y_p^{miss})$. The imputation draws missing values from the predictive distribution $P(Y^{miss}|Y^{obs})$, where, an imputation model with parameter θ is specified and realizations of the predictive distribution of the missing values can be obtained by; drawing θ from $P(\theta|Y^{obs})$ its posterior distribution and drawing missing values according to $P(Y^{miss}|Y^{obs}, \theta)$ their predictive distribution given θ (1). In our case of a single incomplete variable (y_p), the posterior distribution can be specified by letting $\theta = (\beta, \Psi, \sum_k)$ be the parameters of a linear mixed effects model (1):

$$y_{pk} = Z_k\beta + W_k b_k + \varepsilon_k, \qquad (1)$$

$$b_k \sim N(0, \Psi), \qquad (2)$$

$$\varepsilon_k \sim N(0, \textstyle\sum_k) \qquad (3)$$

Where y_{pk} denotes the incomplete variable restricted to the cluster k, $Z_k(n_k \times q)$ and $W_k(n_k \times q')$ are the known covariate arrays corresponding to two subsets of $(y_{1k}, \dots, y(p-1)k)$, β is the q –vector of regression coefficients of fixed effects, b_k is the q'-vector of random effects for cluster k, $\Psi(q' \times q')$ is the between cluster variance matrix, and $\sum_k = \delta_k^2 I_{nk}(n_k \times n_k)$ is the variance matrix within cluster k.

Implementation of the model to DHIS2

Since the number of attendances is count, linear transformation was important before imputation, following an appropriate variance-stabilizing transformation to make the normal distribution assumption more plausible. The variance-stabilizing transformation for the Poisson distribution of count data is the square root, and it provides a better transformation relative to the log transformation for count data (5-9). The back transformed values under the square root method align with the original count scale. A linear mixed effects model was then selected for the implementation of MI. Below is a representation of the specified model in

matrix form:

$$Y_i = X_i\beta + Z_j b_j + \varepsilon_i \quad (4)$$

Where: Y_i is response vector of the indicators X_i the model matrix for the fixed effects (health facility covariates; Health facility ownership (public or private), level of health facility, COVID-19 binary indicator (0 – months before pandemic and 1 – months post pandemic) and time (time data was reported as months and year combined)) and Z_i the model matrix for the random intercept for observations in the jth health facility. The vector of health facility covariates coefficients is represented by β while b_j represents the vector of random-effect coefficients in health facility j . The errors terms denoted by ϵ_i are assumed to follow a multivariate normal distribution with mean vector 0 and variance covariance matrix ∑. The MI mixed effects model was implemented in R version 3.6.3 using the *jomo* package for multilevel imputation (10).

References

1. Audigier V, White IR, Jolani S, Debray TP, Quartagno M, Carpenter J, et al. Multiple imputation for multilevel data with continuous and binary variables. Statistical Science. 2018;33(2):160-83.
2. Quartagno M, Grund S, Carpenter J. Jomo: a flexible package for two-level joint modelling multiple imputation. R Journal. 2019;9(1).
3. Quartagno M, Carpenter J, Quartagno MM, BaBooN S. Package 'jomo'. 2020.
4. Quartagno M, Carpenter J. Multiple imputation for IPD meta-analysis: allowing for heterogeneity and studies with missing covariates. Stat Med. 2016;35(17):2938-54.
5. Quartagno M. Multiple Imputation for Individual Patient Data Meta-Analyses: London School of Hygiene & Tropical Medicine; 2016.
6. Yu G. Variance stabilizing transformations of Poisson, binomial and negative binomial distributions. Statistics & Probability Letters. 2009;79(14):1621-9.
7. Crawley MJ. The R book: John Wiley & Sons; 2012.
8. Maindonald J, Braun J. Data analysis and graphics using R: an example-based approach: Cambridge University Press; 2006.
9. Stroup WW. Rethinking the analysis of non-normal data in plant and soil science. Agron J. 2015;107(2):811-27.
10. Quartagno M, Grund S, Carpenter J. Jomo: a flexible package for two-level joint modelling multiple imputation. R Journal. 2019.
