# Supplementary material for: Quantifying the indirect impact of COVID-19 pandemic on utilisation of outpatient and immunisation services in Kenya: a longitudinal study using interrupted time series analysis
Source: BMJ Open. 2022 Mar 10;12(3):e055815. doi: 10.1136/bmjopen-2021-055815 (PMC8914407; doi:10.1136/bmjopen-2021-055815)
Supplement: Supplementary data [file bmjopen-2021-055815supp003.pdf]

SI Table 1: Number and percentage of health facilities analysed for each indicator. It shows number of facilities that did not report any month and those that were imputed (health facilities with more than 30% of months reported)

Indicator	All hospitals expected to report in DHIS2	Number of health facilities imputed	Number of health facilities with no reported data	Percent of health facilities analysed out of those reporting at least a month
BCG	8063	6509	352	84
DPT1	8063	7142	130	90
DPT2	8063	7141	140	90
DPT3	8063	7136	124	90
IPV	8063	7089	144	90
Measles1	8063	7166	125	90
Measles2	8063	6578	230	84
OPV1	8063	7134	128	90
OPV2	8063	7144	140	90
OPV3	8063	7124	123	90
Pneum1	8063	7139	132	90
Pneum2	8063	7143	141	90
Pneum3	8063	7145	129	90
Rota1	8063	7126	130	90
Rota2	8063	7114	146	90
ANC 1	13595	7768	4450	85
ANC 4	13595	7768	4450	85
OPD > 5 Female	13595	9434	3156	90
OPD > 5 Male	13595	9431	3153	90
OPD < 5 Female	13595	9246	3274	90
OPD < 5 Male	13595	9250	3276	90
OPD Pneumonia > 5	13595	7933	3264	77
OPD Pneumonia < 5	13798	6976	3784	70
Diabetes new cases	13752	72	13472	26
Diabetes total cases	13752	4220	4914	48
Hypertension new cases	13752	121	13454	41
Hypertension total cases	13757	7381	3765	74
HIV tests performed	13752	6789	5674	84
