# Supplementary material for: Quantifying the indirect impact of COVID-19 pandemic on utilisation of outpatient and immunisation services in Kenya: a longitudinal study using interrupted time series analysis
Source: BMJ Open. 2022 Mar 10;12(3):e055815. doi: 10.1136/bmjopen-2021-055815 (PMC8914407; doi:10.1136/bmjopen-2021-055815)
Supplement: Supplementary data [file bmjopen-2021-055815supp004.pdf]

Table 1: Intraclass correlation coefficient (ICC)

Indicator	ICC
OPD <5 years	0.72
OPD > 5 years	0.82
OPD Pneumonia > 5 years	0.52
OPD Pneumonia < 5 years	0.43
ANC 1	0.87
ANC 4	0.76
Diabetes new cases	0.51
Diabetes total cases	0.96
Hypertension new cases	0.93
Hypertension total cases	0.72
HIV tests	0.92

Table 2: Akaike Information Criterion (AIC)

Indicator	Negative Binomial model	Negative Binomial model accounting for seasonality
OPD <5 years	1061.42	1042.73
OPD > 5 years	1147.58	1138.40
OPD Pneumonia < 5 years	841.78	832.94
OPD Pneumonia > 5 years	849.94	846.07
ANC 1	858.49	857.58
ANC 4	791.49	763.62
Diabetes new cases	419.34	417.93
Diabetes total cases	731.45	730.82
Hypertension new cases	340.09	338.17
Hypertension total cases	830.11	819.39
HIV tests	1008.38	1005.78

Table 3: Segmented regression estimates for Negative binomial model before adjusting for seasonality

Covariate	OPD < 5 years			OPD > 5 years			OPD Pneumonia < 5 years			OPD Pneumonia > 5 years		
	RR	95%CI	P-value	RR	95%CI	P-value	RR	95%CI	P-value	RR	95%CI	P-value
COVID-19	0.56	(0.47-0.65)	<0.01	0.72	(0.62-0.84)	<0.01	0.49	(0.42-0.57)	<0.01	0.69	(0.59-0.82))	<0.01
Time	1.00	(1.00-1.01)	0.75	1.00	(1.00-1.01)	0.18	1.01	(1.00-1.01)	<0.01	1.00	(1.00-1.01)	0.46
Trend	1.04	(1.02-1.06)	<0.01	1.01	(0.99-1.03)	0.42	1.03	(1.01-1.05)	<0.01	1.00	(1.00-1.04)	0.05
	ANC 1			ANC 4			Diabetes new cases			Diabetes total cases		
	RR	95%CI	P-value	RR	95%CI	P-value	RR	95%CI	P-value	RR	95%CI	P-value
COVID-19	0.96	(0.83-1.10)	0.53	0.95	(0.84-1.08)	0.45	1.12	(0.89-1.41)	0.34	0.97	(0.95-0.98)	<0.01
Time	1.00	(0.99-1.00)	0.45	1.00	(1.00-1.01	0.52	0.99	(0.98-1.00)	0.14	1.01	(1.00-1.02)	<0.01
Trend	1.02	(1.00-1.03)	0.07	0.99	(0.97-1.01)	0.25	1.00	(0.99-1.02)	0.87	1.00	(0.99-1.00)	0.56
	Hypertension new cases			Hypertension total cases			HIV Tests Performed					
	RR	95%CI	P-value	RR	95%CI	P-value	RR	95%CI	P-value			
COVID-19	0.92	(0.82-1.04)	0.21	0.92	(0.86-0.99)	0.03	1.01	(0.98-1.05)	0.41			
Time	0.99	(0.99-1.01)	0.18	1.01	(1.00-1.01)	<0.01	0.97	(0.97-0.97)	<0.01			
Trend	1.04	(0.99-1.10)	0.14	0.99	(0.98-1.00)	<0.01	1.00	(0.99-1.01)	0.69			
