# Supplementary material for: Quantifying the indirect impact of COVID-19 pandemic on utilisation of outpatient and immunisation services in Kenya: a longitudinal study using interrupted time series analysis
Source: BMJ Open. 2022 Mar 10;12(3):e055815. doi: 10.1136/bmjopen-2021-055815 (PMC8914407; doi:10.1136/bmjopen-2021-055815)
Supplement: Supplementary data [file bmjopen-2021-055815supp005.pdf]

SI Figure 1: Multiple change point analysis plots showing significant shifts in attendance

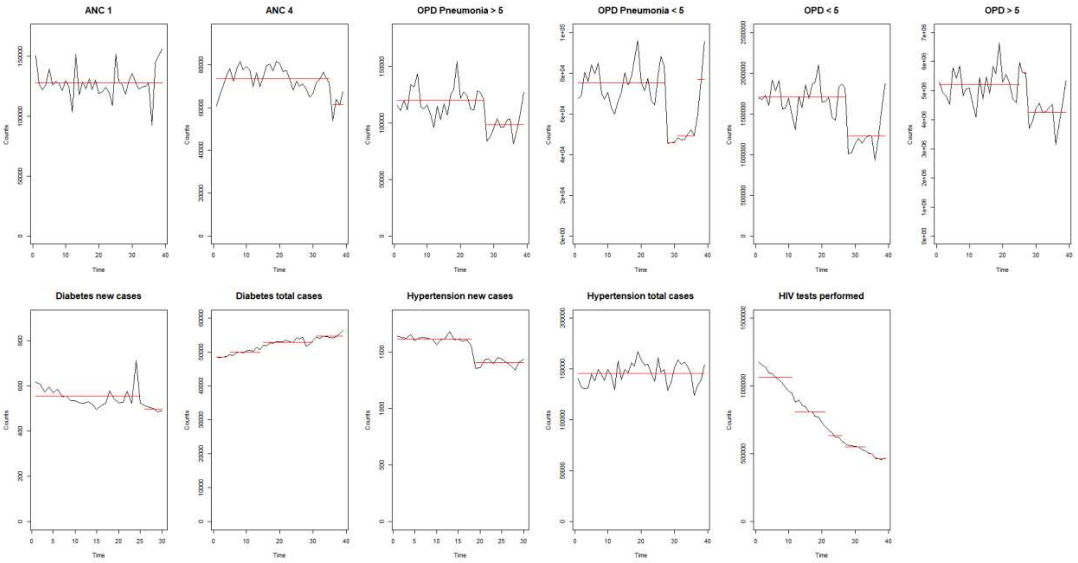

SI Table 1: Interrupted time series models comparing estimates before and after excluding strike period

		OPD < 5			OPD > 5			OPD Pneumonia < 5			OPD Pneumonia > 5		
Ownership		RR	95%CI	P-value	RR	95%CI	P-value	RR	95%CI	P-value	RR	95%CI	P-value
Primary	COVID-19	0.50	(0.44-0.57)	<0.01	0.65	(0.57-0.75)	<0.01	0.43	(0.38-0.47)	<0.01	0.62	(0.55-0.70)	<0.01
	Time	1.00	(0.99-1.01)	0.15	1.00	(1.00-1.01)	0.02	1.01	(1.00-1.01)	<0.01	1.00	(0.99-1.01)	0.05
	Trend	1.05	(1.03-1.06)	<0.01	1.02	(1.00-1.04)	0.03	1.07	(1.05-1.08)	<0.01	1.03	(1.02-1.05)	<0.01
Excluding Strike	COVID-19	0.45	(0.39-0.52)	<0.01	0.60	(0.53-0.68)	<0.01	0.39	(0.33-0.47)	<0.01	0.58	(0.52-0.66)	<0.01
	Time	1.00	(1.00-1.01)	0.13	1.01	(1.00-1.01)	<0.01	1.01	(1.00-1.01)	0.02	1.00	(1.00-1.01)	0.03
	Trend	1.09	(1.06-1.11)	<0.01	1.05	(1.03-1.07)	<0.01	1.10	(1.07-1.13)	<0.01	1.06	(1.04-1.08)	<0.01
		ANC 1			ANC 4			Diabetes new cases			Diabetes total cases		
		RR	95%CI	P-value	RR	95%CI	P-value	RR	95%CI	P-value	RR	95%CI	P-value
Primary	COVID-19	0.96	(0.83-1.10)	0.55	0.86	(0.80-0.93)	<0.01	1.17	(0.89-1.52)	0.25	0.95	(0.93-0.97)	<0.01
	Time	1.00	(0.99-1.00)	0.61	1.00	(0.99-1.00)	0.13	0.99	(0.98-1.00)	0.13	1.00	(1.00-1.01)	<0.01
	Trend	1.01	(0.99-1.03)	0.12	1.00	(0.99-1.01)	0.90	0.99	(0.97-1.01)	0.57	1.00	(1.00-1.00)	0.05
Excluding Strike	COVID-19	0.96	(0.84-1.09)	0.52	0.83	(0.77-0.89)	<0.01	1.12	(0.85-1.48)	0.43	0.94	(0.92-0.96)	<0.01
	Time	1.00	(1.00-1.00)	0.44	1.00	(1.00-1.00)	0.05	0.99	(0.98-1.00)	0.12	1.00	(1.00-1.01)	<0.01
	Trend	1.02	(1.00-1.04)	0.06	1.01	(1.00-1.03)	0.02	1.01	(0.98-1.04)	0.73	1.01	(1.00-1.01)	<0.01
		Hypertension new cases			Hypertension total cases			HIV Tests Performed					
		RR	95%CI	P-value	RR	95%CI	P-value	RR	95%CI	P-value			
Primary	COVID-19	0.87	(0.75-1.00)	0.05	0.89	(0.82-0.96)	<0.01	0.97	(0.94-0.99)	0.01			
	Time	1.00	(0.99-1.01)	0.81	1.01	(1.00-1.01)	<0.01	0.97	(0.97-0.97)	<0.01			
	Trend	1.00	(0.99-1.01)	0.59	1.00	(0.99-1.01)	0.90	1.01	(1.01-1.01)	<0.01			
Excluding Strike	COVID-19	0.86	(0.74-1.00)	0.06	0.85	(0.79-0.92)	<0.01	0.97	(0.94-1.01)	0.11			
	Time	1.00	(0.99-1.01)	0.81	1.01	(1.00-1.01)	<0.01	0.97	(0.97-0.97)	<0.01			
	Trend	1.01	(0.99-1.02)	0.48	1.01	(1.00-1.03)	0.02	1.01	(1.00-1.01)	<0.01			

SI Table 2: Generalised estimating equations (GEE) results at health facility level showing rate ratios (RR) for COVID-19 intervention, time and trend alongside 95% confidence intervals for all indicators

		OPD < 5			OPD > 5			OPD Pneumonia < 5			OPD Pneumonia > 5		
**Ownership**		**RR**	**95%CI**	**P-value**	**RR**	**95%CI**	**P-value**	**RR**	**95%CI**	**P-value**	**RR**	**95%CI**	**P-value**
**Primary**	**COVID-19**	0.50	(0.44-0.57)	<0.01	0.65	(0.57-0.75)	<0.01	0.43	(0.38-0.47)	<0.01	0.62	(0.55-0.70)	<0.01
	**Time**	1.00	(0.99-1.01)	0.15	1.00	(1.00-1.01)	0.02	1.01	(1.00-1.01)	<0.01	1.00	(0.99-1.01)	0.05
	**Trend**	1.05	(1.03-1.06)	<0.01	1.02	(1.00-1.04)	0.03	1.07	(1.05-1.08)	<0.01	1.03	(1.02-1.05)	<0.01
**GEE**	**COVID-19**	0.50	(0.48-0.51)	<0.01	0.65	(0.64-0.66)	<0.01	0.42	(0.40-0.43)	<0.01	0.62	(0.60-0.64)	<0.01
	**Time**	1.00	(1.00-1.01)	<0.01	1.01	(1.00-1.01)	<0.01	1.01	(1.00-1.01)	<0.01	1.00	(1.00-1.01)	<0.01
	**Trend**	1.05	(1.04-1.05)	<0.01	1.02	(1.01-1.02)	<0.01	1.07	(1.06-1.07)	<0.01	1.03	(1.03-1.04)	<0.01
		**ANC 1**			**ANC 4**			**Diabetes new cases**			**Diabetes total cases**		
		**RR**	**95%CI**	**P-value**	**RR**	**95%CI**	**P-value**	**RR**	**95%CI**	**P-value**	**RR**	**95%CI**	**P-value**
**Primary**	**COVID-19**	0.96	(0.83-1.10)	0.55	0.86	(0.80-0.93)	<0.01	1.17	(0.89-1.52)	0.25	0.95	(0.93-0.97)	<0.01
	**Time**	1.00	(0.99-1.00)	0.61	1.00	(0.99-1.00)	0.13	0.99	(0.98-1.00)	0.13	1.00	(1.00-1.01)	<0.01
	**Trend**	1.01	(0.99-1.03)	0.12	1.00	(0.99-1.01)	0.90	0.99	(0.97-1.01)	0.57	1.00	(1.00-1.00)	0.05
**GEE**	**COVID-19**	0.96	(0.92-0.99)	0.01	0.87	(0.83-0.90)	<0.01	1.17	(0.89-1.53)	0.26	0.95	(0.93-0.98)	<0.01
	**Time**	1.00	(0.99-1.00)	0.05	1.00	(1.00-1.01)	0.01	0.99	(0.98-1.00)	0.13	1.00	(1.00-1.01)	<0.01
	**Trend**	1.01	(1.01-1.02)	<0.01	1.00	(0.99-1.01)	0.71	0.99	(0.98-1.01)	0.58	1.00	(1.00-1.00)	0.26
		**Hypertension new cases**			**Hypertension total cases**			**HIV Tests Performed**					
		**RR**	**95%CI**	**P-value**	**RR**	**95%CI**	**P-value**	**RR**	**95%CI**	**P-value**			
**Primary**	**COVID-19**	0.87	(0.75-1.00)	0.05	0.89	(0.82-0.96)	<0.01	0.97	(0.94-0.99)	0.01			
	**Time**	1.00	(0.99-1.01)	0.81	1.01	(1.00-1.01)	<0.01	0.97	(0.97-0.97)	<0.01			
	**Trend**	1.00	(0.99-1.01)	0.59	1.00	(0.99-1.01)	0.90	1.01	(1.01-1.01)	<0.01			
**GEE**	**COVID-19**	0.87	(0.74-1.02)	0.09	0.89	(0.85-0.92)	<0.01	0.96	(0.95-0.98)	<0.01			
	**Time**	1.00	(0.99-1.01)	0.83	1.01	(1.00-1.01)	<0.01	0.97	(0.97-0.97)	<0.01			
	**Trend**	1.00	(0.99-1.02)	0.70	1.00	(1.00-1.01)	0.83	1.01	(1.01-1.01)	<0.01			
