# Supplementary material for: Quantifying the indirect impact of COVID-19 pandemic on utilisation of outpatient and immunisation services in Kenya: a longitudinal study using interrupted time series analysis
Source: BMJ Open. 2022 Mar 10;12(3):e055815. doi: 10.1136/bmjopen-2021-055815 (PMC8914407; doi:10.1136/bmjopen-2021-055815)
Supplement: Author's manuscript [file bmjopen-2021-055815.draft_revisions.pdf]

BMJ Open is committed to open peer review. As part of this commitment we make the peer review history of every article we publish publicly available.

When an article is published we post the peer reviewers' comments and the authors' responses online. We also post the versions of the paper that were used during peer review. These are the versions that the peer review comments apply to.

The versions of the paper that follow are the versions that were submitted during the peer review process. They are not the versions of record or the final published versions. They should not be cited or distributed as the published version of this manuscript.

BMJ Open is an open access journal and the full, final, typeset and author-corrected version of record of the manuscript is available on our site with no access controls, subscription charges or pay-per-view fees (http://bmjopen.bmj.com).

If you have any questions on BMJ Open's open peer review process please email info.bmjopen@bmj.com

BMJ Open

Quantifying the indirect impact of COVID-19 pandemic on utilisation of outpatient and immunisation services in Kenya: An interrupted time series analysis

Journal:	*BMJ Open*
Manuscript ID	bmjopen-2021-055815
Article Type:	Original research
Date Submitted by the Author:	24-Jul-2021

Complete List of Authors:	Wambua, Steven; KEMRI-Wellcome Trust Research Programme Nairobi, Population Health Unit<br>Malla, Lucas; KEMRI-Wellcome Trust Research Programme Nairobi, Health Services Unit<br>Mbevi, George; KEMRI-Wellcome Trust Research Programme Nairobi, Health Services Unit<br>Kandiah, Joel; University of Warwick, Mathematics Institute<br>Nwosu, Amen-Patrick; Oxford Centre for Global Health Research, Nuffield Department of Clinical Medicine<br>Tuti, Timothy; KEMRI-Wellcome Trust Research Programme Nairobi, Health Services Unit<br>Paton, Chris; Oxford Centre for Global Health Research, Nuffield Department of Clinical Medicine<br>Wambu, Bernard; Kenya Ministry of Health<br>English, Mike; KEMRI-Wellcome Trust Research Programme Nairobi, Health Services Unit; Oxford Centre for Global Health Research, Nuffield Department of Clinical Medicine<br>Okiro, Emelda ; KEMRI-Wellcome Trust Research Programme Nairobi, Population Health Unit; Oxford Centre for Global Health Research, Nuffield Department of Clinical Medicine

Keywords:	Public health < INFECTIOUS DISEASES, COVID-19, Paediatric infectious disease & immunisation < PAEDIATRICS, International health services < HEALTH SERVICES ADMINISTRATION & MANAGEMENT

SCHOLARONE™
Manuscripts

I, the Submitting Author has the right to grant and does grant on behalf of all authors of the Work (as defined in the below author licence), an exclusive licence and/or a non-exclusive licence for contributions from authors who are: i) UK Crown employees; ii) where BMJ has agreed a CC-BY licence shall apply, and/or iii) in accordance with the terms applicable for US Federal Government officers or employees acting as part of their official duties; on a worldwide, perpetual, irrevocable, royalty-free basis to BMJ Publishing Group Ltd ("BMJ") its licensees and where the relevant Journal is co-owned by BMJ to the co-owners of the Journal, to publish the Work in this journal and any other BMJ products and to exploit all rights, as set out in our licence.

The Submitting Author accepts and understands that any supply made under these terms is made by BMJ to the Submitting Author unless you are acting as an employee on behalf of your employer or a postgraduate student of an affiliated institution which is paying any applicable article publishing charge ("APC") for Open Access articles. Where the Submitting Author wishes to make the Work available on an Open Access basis (and intends to pay the relevant APC), the terms of reuse of such Open Access shall be governed by a Creative Commons licence – details of these licences and which Creative Commons licence will apply to this Work are set out in our licence referred to above.

Other than as permitted in any relevant BMJ Author's Self Archiving Policies, I confirm this Work has not been accepted for publication elsewhere, is not being considered for publication elsewhere and does not duplicate material already published. I confirm all authors consent to publication of this Work and authorise the granting of this licence.

For peer review only

Quantifying the indirect impact of COVID-19 pandemic on utilisation of outpatient and immunisation services in Kenya: An interrupted time series analysis

Steven Wambua[1]*, Lucas Malla[2], George Mbevi[2], Joel Kandiah[4], Amen-Patrick Nwosu[3], Timothy Tuti[2], Chris Paton[3], Bernard Wambu[5], Mike English[2,3], Emelda A. Okiro[1,3].

[1]Population Health Unit, Kenya Medical Research Institute-Wellcome Trust Research Programme, Nairobi, Kenya

[2]Health Services Unit, KEMRI-Wellcome Trust Research Programme, Nairobi, Kenya

[3]Oxford Centre for Global Health Research, Nuffield Department of Clinical Medicine, University of Oxford, Oxford, U.K.

[4]University of Warwick, Mathematics Institute, Coventry, U.K

[5]Ministry of Health, Nairobi, Kenya

*Corresponding author

SW*:swambua@kemri-wellcome.org, PO BOX 43640-00100, Nairobi Kenya, Tel: 0718618560

For peer review only

Word Count: 3952

For peer review only

Abstract

Objective: In this study we assess the indirect impact of COVID-19 on utilization of immunisation and outpatient services in Kenya.

Design: Longitudinal study

Setting: Data were analysed from all healthcare facilities reporting to Kenya's health information system from January 2018 to March 2021.

Exposure of interest: COVID-19 outbreak and associated interventions

Outcome measures: Monthly attendance to health facilities. We assessed changes in immunization and various outpatient services nationally.

Results

Before the first case of COVID-19 and pursuant intervention measures in March 2020, uptake of health services was consistent with historical levels. There was significant drops in attendance (level changes) in April 2020 for overall outpatient visits for under-fives (50%), under-fives with pneumonia (43%), overall over-five visits (35%), over-fives with pneumonia (38%), fourth antenatal care visit (14%), total hypertension (11%), diabetes cases (5%) and HIV testing (3%). Immunization services, first antenatal care visits, new cases of hypertension and diabetes were not affected. The post-COVID-19 trend was increasing, with more recent data suggesting reversal of effects and health services reverting to expected levels as of March 2021.

Conclusion

COVID-19 pandemic has had varied indirect effects on utilization of health services in Kenya. There is need for proactive and targeted interventions to reverse these effects as part of the pandemic's response to avert non-COVID-19 indirect mortality.

Keywords: COVID-19, SARS-CoV-2, outpatient services, immunization, DHIS2

Strengths and limitations of this study

For peer review only

- This analysis is strengthened by use of a broad set of health services indicators and over a large number of health facilities nationally and a longer time period (39 months) allowing for the adjustment of pre-COVID-19 trends.
- We have adjusted for factors such as health workers strikes and missing data in the analysis strengthening the validity of the results.
- Data was analysed across the whole health system in Kenya (both public and private sector) therefore can be used to predict impact in other similar settings.
- COVID-19 outbreak and associated public health measures were not random. Other concurrent unmeasured factors or shocks could have contributed, however small, to the changes.
- This study doesn't allow for in-depth evaluation of the specific causes of the trends observed within a qualitative framework because it was purely quantitative.

For peer review only

Introduction

The novel coronavirus (COVID-19) outbreak was declared a global pandemic by the World Health Organization (WHO) on March 11, 2020. By 6th May 2021, 156 million cases and 3.2 million deaths have been reported globally,[1]. Since the first case of COVID-19 was reported in Kenya on 13th March 2020, 162,098 cases and 2850 deaths were reported by 6th May 2021,[1]. The government, in attempt to control the spread of the pandemic, instituted a raft of interventions. Consequently, beyond the pandemic's direct impact on the population health, indirect effects due to the control measures, changes in public and clinician behaviour and health system reorganization are likely to manifest in changes to utilisation of essential health services.

The country has experienced three waves of the pandemic,[2]. The first wave peaked in July/August 2020,[3]. During this wave, the government suspended all public gatherings, closed schools and bars, limited restaurants to take-aways, reduced transport capacity , was announced a national dusk-to-dawn curfew, suspended international flights, mandated face masks in public, and the four counties (Nairobi, Mombasa, Kwale and Kilifi) with the highest

number of cases were put under lockdown, with cessation of movement to other neighbouring countries. Some of these restrictions were relaxed between July 6th and 1st August 2020. During the second wave, which peaked in November 2020,[3], there was phased reopening of schools and universities and suspension of political gatherings. In January 4th, 2021, all schools were reopened. In March 5th2021, the COVID-19 vaccination campaign targeting 1.02 million health workers and those above the age of 58 years was launched,[4]. In March 2021, the country experienced the third wave with the highest daily cases recorded since the start of pandemic.

The public health interventions are expected to have economic and social impacts such as reductions in manufacturing, access to employment and basic necessities,[5, 6]. Consequently, access and utilisation of essential health services are likely to be affected,[7]. Early modelled predictions showed reductions in utilization of health services,[8, 9]. In addition, studies during previous epidemics in sub-Saharan Africa reported a reduction in utilisation of essential health services during and after outbreaks,[10-14]. Various population groups are likely to be affected differently, with children and women at a higher risk,[10, 15]. These interruptions in health service utilisation are raising concerns of increased morbidity and mortality for non-COVID-19 illnesses and especially for childcare services,[9]. Although recent studies have reported variable impact of the pandemic on various health services, the impact

For peer review only

on administration of vaccines and monitoring a broad set of essential services over a longer - observation period after the pandemic was announced by WHO has not been evaluated rigorously in Kenya,[16-19].

Using the Kenya's routine health information system implemented through the District Health Information Software version 2 (DHIS 2), a database where all health facilities in Kenya are expected to report services they offered in a given month, this study aimed to assess the indirect impact of COVID-19 on utilisation of varied basic essential health services nationally.

Methods

Timeline of events

Pre-COVID-19 measures

Two months before the first case of COVID-19 was reported in Kenya, the government increased preparedness towards the pandemic. These included installation of surveillance systems to detect suspected COVID-19 cases at border points, additional medical staff at international airports and ports, in-country capacity to test and isolate COVID-19 cases, sensitisation of healthcare workers on dealing with COVID-19 cases and establishment of a National Emergency Response Committee.

For peer review only

Post-COVID-19 control measures

The government started the introduction of interventions to combat COVID-19 spread on 13th March 2020. These included suspension of public gatherings and events, closing of schools, international travel restrictions, fumigation and disinfection of markets, closure of bars and restaurants, suspension of attendance to places of worship, limit of people attending weddings and funerals and a national dust-to-dawn curfew. The month of April 2020 saw cessation of movement in and out of four counties with highest number of COVID-19 cases, restaurants were opened under strict guidelines of social distancing, handwashing and temperature checks. During the month of May 2020, cessation of movement into and out of Kenya through Tanzania and Somalia borders was affected while in June 2020 the government launched home-based care for patients with COVID-19 infection. In July 2020, certain measures were relaxed: cessation of movement into the four counties was lifted, phased re-opening of places of worship, and resumption of local air travel. In August 2020, international air travel resumed and in September 2020, operation of bars resumed. This was followed by phased re-opening of schools and lifting of suspension on political gatherings in October 2020 and November 2020 respectively. Between December 2020 to February 2021 there was a national health

For peer review only

workers strike triggered by demands for better working conditions such as provision of adequate Personal Protective Equipment (PPE), enhanced risk allowances and a health insurance cover. Although the length of the strike varied by health facilities and cadre of health workers, we couldn't obtain a database which tracks strikes nationally, and we therefore assumed most of the health facilities were on strike during the whole period. All schools re-opened in January 2021. The timeline of events is presented in Figure 1.

-------------------------------------Figure 1---

For peer review only

Data

We extracted monthly data from DHIS 2 for the period January 2018 to March 2021 on total outpatient visits (under and over-fives), the number of hypertension and diabetes cases and HIV tests performed, doses of immunisation antigens administered and antenatal care visits (the first (ANC 1) and fourth (ANC 4) visits). A description of the indicators is presented in Table 1.

---Table 1--

Data were not available for the period January 2018 to September 2018 for hypertension and diabetes new cases. For both indicators and for relevant periods data were excluded from the analysis. We chose 2018 as a starting point because of prolonged health care worker strikes in 2017 which affected health services provision,[20] and consequently reporting. Data were cleaned to remove duplicated health facilities. Extreme outliers, defined as values that are more than 3 standard deviations from the mean of reported values for a given health facility,[21, 22], were identified, investigated and treated as missing. For each health facility, we obtained the administrative units, level of the facility (Level 2: Dispensaries with outpatient services only, Level 3: Comprehensive primary health care facilities, Level 4: primary referral hospitals, Level 5: Secondary referral hospitals and Level 6: national teaching and referral hospitals) and whether the health facility is private or public.

For peer review only

Statistical analysis

Handling missing data

To adjust for incompleteness in reporting, multiple imputation was performed,[23-25]. Missing monthly values were imputed using a mixed effects model in a joint modelling framework,[26, 27]. Health facility ownership (public or private), level of health facility, time (month and year) and COVID-19 binary indicator (0 – months before pandemic and 1 – months post pandemic) were used as covariates with the health facility as a clustering variable. MI was performed for

health facilities with more than 30% of months reported (at least 12 months reported) to reduce uncertainty in imputed values and ensure generalizability of the estimates. Additionally, through a simulation study we found MI performance and efficiency was best when imputing for health facilities with more than 30% of months reported. The number of health facilities analysed is presented in Additional File 1 SI Table 1.

Interrupted time series analysis

Exploratory analyses

Data were aggregated monthly for all health facilities. Trends were plotted to visualise changes in utilisation of health services. Statistical process control (SPC) charts with the 2018-2019 average as a baseline were used to identify significant shifts in monthly values for 2020-2021. Values that are more than 3 standard deviations from the mean are considered significant shifts and were carried forward for interrupted time series analysis [28]. Multiple change point analysis was applied to assess the influence of health worker strike on provision of health services,[29, 30].

Segmented regression

We conducted interrupted time series analyses using monthly attendance counts for each indicator as outcomes. The period running from January 2018 through March 2020 when the

For peer review only

first case was identified was defined as pre-COVID-19 and April 2020 to March 2021 as post-COVID-19. For indicators where changes were observed in SPC analysis, segmented regression were performed to model attendance before and after COVID-19 was reported,[31, 32]. The following equation specifies the model,[31];

$$Y_t = \beta_0 + \beta_1 * time_t + \beta_2 * COVID19_t + \beta_3 * time\ after\ COVID19_t + e_t$$

Where, Y_t is the attendance in month t; $time$ is a continuous indicator of time in months from January 2018; $COVID19$ is an indicator of time t occurring before ($COVID19 = 0$) or after ($COVID19 = 1$) the outbreak, which was implemented at April 2021 in the series; and $time\ after\ COVID19$ is a continuous variable of the number of months after COVID-19 at time t. In the model, β_0 estimates the baseline level of attendance at time zero; β_1 estimates the change in monthly number of visits before COVID-19 (pre-existing trend); β_2 estimates the level change immediately after COVID-19 outbreak; β_3 estimates the change in the trend after COVID-19, compared with the pre-existing trend. A change in intercept (immediate COVID-19 effect) and change in slope (gradual COVID-19 effect) were hypothesised,[32].

A generalised linear model was applied assuming a Poisson distribution. We fitted both Poisson and Negative binomial models to account for over-dispersion,[32-34]. Model performance was evaluated using the Akaike's information criterion,[35]. Model checking was conducted for autocorrelation using the Durbin-Watson statistic and autoregressive moving

For peer review only

average (ARMA) models were fitted for indicators with serial autocorrelation,[36-38]. Seasonality was adjusted using Fourier terms,[39]. Results were pooled across the multiple imputed datasets using Rubin's rules,[40]. The negative binomial model, which was adjusted for seasonality and autocorrelation was the best fitting model and it's results are presented in this study.

As a form of sensitivity analysis, we fitted models excluding months when the national strike occurred and compared estimates with those where data included the strike. We also fitted health-facility level generalised estimating equations to test the impact of varying model assumptions on the primary model estimates and hence evaluate robustness of our results,[32].

Statistical significance was defined as p-values < 0.05. All analyses were performed using R (version 3.6.3).

Patient and public involvement

No patients were involved in this study. We have used secondary aggregated routine health information data available online.

For peer review only

Results

COVID-19 impact

------------------------------------Figure 2--

Annual trends show the first antenatal care visits remained unaffected while the fourth visits experienced a downward trend from March 2020. Immunization services remained unaffected with observed spikes in administration of measles vaccines in March 2020. Utilization of outpatient services (overall and due to pneumonia) by under-fives experienced drops after March 2020. Reductions were also experienced in over-fives attendance, hypertension cases and diabetes attendance. HIV testing experienced a gradual decline over the years (Figure 2).

------------------------------------Figure 3---

Further, SPC charts confirmed significant reductions (less than 3SD) in ANC 4 starting April 2020. Immunization services remained unaffected during the same period, with significant increase (more than 3SD) in measles vaccination in March 2020. Moreover, significant reductions in under-fives attendance, over-fives attendance and new visits by hypertensive patients were observed starting April 2020 with no significant reductions for HIV testing and diabetes visits (Figure 3). Additionally, utilization of most services reduced the most in

For peer review only

December 2020 coinciding with start of health care workers strike, after which utilization of most services started to go back to expected levels.

--Table 2--

We fitted interrupted time series models for indicators that showed significant changes from the SPC charts. The rate ratios from the model are presented in Table 1. The month-to-month changes before COVID-19 were generally increasing across all the indicators. There was an immediate statistically significant reduction in all the indicators post-COVID-19, in the month immediately after first case, except for ANC 1 and new cases of diabetes and hypertension, which were unaffected. The statistically significant level changes post-COVID-19 were outpatient attendance for children under-fives which reduced by 50%, those for outpatients' over-fives by 35%, under-fives pneumonia outpatients by 43%, over-fives pneumonia outpatients by 38%, antenatal care 4th visit by 14%, total cases of diabetes by 5%, new cases of hypertension by 11% and HIV tests by 3%. There was a slight but statistically significant month-to-month increase in services post-COVID-19 (April 2020 to March 2021) of 5% for under-fives outpatients attendance, 2% for over-fives outpatients, 4% for under-fives pneumonia outpatients, 3% for over-fives pneumonia patients and no significant month-to-month changes for antenatal care visits, diabetes and hypertension cases. The trends from the fitted interrupted time series model are visually represented in Figure 4.

For peer review only

------------------------------------Figure 4--

Sensitivity analyses

Change point analysis showed the health workers' strike, which started in December 2020 had a significant impact on antenatal care 4th visits, and no effect on the other indicators (Additional File 3 SI Figure 1). Further, excluding the strike period (December 2020 to February 2021) from the segmented regression models of all indicators evaluated resulted in estimates that are not different from primary model estimates (Additional File 3 SI Table 1). Estimates from the Generalised estimating equations (GEE) models were not different from the primary model indicating robustness of reported estimates (Additional File 3 SI Table 2).

Discussion

Using DHIS2 health facility level monthly reported outpatient data, we provide evidence of COVID-19 impact on utilisation of basic health services in Kenya. The announcement of the first case of COVID-19 in Kenya in March 2020 and the intervention measures that followed coincided with sharp declines in outpatient and antenatal care fourth visits nationally. By the end of this study, health services are still in the process of returning to pre-COVID-19 levels. However, immunisation services remained unaffected.

For peer review only

Previous studies have found variable impacts on immunisation services,[17, 41, 42]. In two studies that evaluated performance of routine immunization on selected indicators in Kenya, which used a relatively shorter period and didn't account for missing data, COVID-19 had no substantial impact on vaccination coverage, antenatal care first visits and a significant increase in measles immunization in March 2020 was reported,[17, 41]. The significant increase in measles vaccines in March 2020 was due to increased immunization to make up for stock-out of measles vaccines between November 2019 and January 2020,[17]. The sustained immunisation levels in the other antigens suggests there were no significant disruption to vaccine supply chain resulting from the pandemic, and confirmed by the National Vaccines and Immunisation Programme (NVIP),[17]. Additionally, where health facilities designated as vaccination centres were assigned as COVID-19 isolation centres, the vaccines programme moved immunisation services to neighbouring health facilities,[17]. These strategies illuminate why immunisation services remained unaffected during the pandemic, contrary to earlier predictions of reductions in immunization,[8, 9]. Although not statistically significant, the slight reductions in the number of vaccines administered in December 2020 were likely attributed to the nationwide health worker strike, which led to staff shortages consequently affecting administration of the vaccines. These results strengthen previous findings with no observable differences in mean monthly number of immunisation and total

For peer review only

antenatal care visits over a much shorter study period March-June 2020 relative to the same period in 2019 in Kenya,[42]. In summary, immunisation services were unaffected likely because of a number of reasons; the concerted effort by the NVIP to sustain supply of vaccines and unavailability of alternative sources for vaccination outside of the health system.

There were significant drops in nearly all outpatient services evaluated in this study. Total outpatient and pneumonia specific outpatient attendance were most affected, with utilization of the services dropping by half for under-fives. Moreover, COVID-19 had an impact on ANC 4, total attendance for hypertension and diabetes and HIV testing. Similar findings have been reported in other low- and middle-income countries,[16, 17, 19, 43-46]. Studies evaluating the impact of lockdown measures to combat COVID-19 in South Africa observed a substantial drop in primary healthcare services utilisation,[16, 45]. Significant drops in essential health services were also experienced following institution of public health measures to combat COVID-19 in Kinshasa, Democratic Republic of Congo,[19]. Disruptions in general attendance have also been reported in various studies globally,[43, 47-50].

Various factors could explain the downward trends in specific outpatient services. In a survey conducted in Kenya to assess health services utilization during COVID-19, common causes reported by respondents include fear of risk of catching coronavirus at health facilities (26%), reduced incomes affecting ability to meet transport costs and other healthcare related costs

For peer review only

(17%), shortage of healthcare workers in health facilities (14%), difficulties in accessing health facilities due to lockdowns and curfew (14%) and closing of some health facilities (14%),[51]. The substantial declines for under-fives attendance are likely associated with reduced mixing due to closure of schools, improved hygiene practices and parents choosing to manage non-severe illnesses at home. Although attendance for ANC 4 was affected, it is unclear why the first visits were not affected. Notwithstanding, this might suggest that pregnant women attach greater importance to the primary ANC visit as has been reported,[52, 53] and hence despite the prevailing conditions managed to prioritize at least one visit to a health facility. Additionally, data has suggested deliveries in health facilities were also not affected during the pandemic (Wambua et al 2021, The indirect impact of COVID-19 pandemic on inpatient admissions in 204 Kenyan hospitals: An interrupted time series analysis), and this likely suggests the population of pregnant women remained relatively comfortable to use health services despite the pandemic.

A survey in Ethiopia among diabetic and hypertensive patients reported unavailability, unaffordable or increased price of medications and interruptions in follow-up visits were common barriers to accessing chronic care units in public facilities during the pandemic,[54]. Reduction in attendance for chronic conditions such as hypertensive cases is a significant finding as missing care for these chronic illnesses could lead to further complications and

For peer review only

susceptibility to severe COVID-19,[55] and increased morbidity and mortality. The gradual decline in HIV testing pre- COVID-19 might suggest reduced coverage due to policies geared towards targeted testing as opposed to blanket testing,[56]. Additionally increased uptake and accessibility to testing in pharmacies implemented in 2017 might be associated with reduced testing in health facilities,[57]. Pre-existing challenges in access to health services such as poor road network, disruptions in supplies to health facilities, and limited or no capacity for domestic production of medical supplies could have compounded the dramatic downward trends in utilisation of outpatient services. Additionally, improved hand hygiene and use of face masks during the pandemic could have led to reduced risk of other infectious diseases and consequently fewer visits to health facilities,[58, 59].

Strengths and implications of the study

Although most of the public attention is on control measures of COVID-19, possible health consequences from the indirect effects of the measures should not be overlooked. We provide a comprehensive understanding of the present situation on utilisation of immunisation and outpatient services in Kenya. Although the findings provide short-term estimates on the effect of COVID-19 at national level, studies could assess the long-term and differential effects at sub-national level. We addressed possible confounders in assessing changes overtime. For instance, in line with a recent guide on using routine data to monitor the effects of COVID-19

For peer review only

by the WHO, we adjusted for missing data which would have affected the validity of the comparisons over time,[60]. Additionally, incompleteness may lead to biased estimates and strategies to improve data quality in DHIS2 such as investment in better infrastructure, supervisory support, formal data quality assurance and human resources could improve reporting in Kenyan health facilities,[61, 62]. We also use sensitivity analysis to account for any uncertainty in the estimates due to other factors affecting utilisation of services such as healthcare workers strikes and health-facility specific variations, which reduced bias and improved precision of the estimates.

For peer review only

Limitations

In this study, controls were not used to differentiate the impact of COVID-19 from other possible causes of the changes as most indicators were indirectly affected by the pandemic. However, since the drops in utilisation of services coincided with the introduction of COVID-19 intervention measures, the changes are attributed to COVID-19.

Conclusion

In summary, COVID-19 pandemic has had varied indirect effects on utilisation of outpatient health services. Although utilisation of immunisation services remained unchanged, there was

a significant negative impact on outpatient clinic and ANC visits nationally. Total outpatient attendances for children under-fives reduced by 50%, under-fives pneumonia presentations reduced by 50%, general over-five visits reduced by 35%, over-fives pneumonia reduced by 38%, ANC 4 visits reduced by 14%, total hypertension cases reduced by 11%,total diabetes cases reduced by 5% and HIV testing by 3%. There is need for proactive and targeted interventions to avert and reverse these effects in future pandemics. These include strict implementation of safe practices and infrastructural changes in health facilities to reassure the public that it's safe to go to health facilities. Other innovative measures such as safe modes of transport, mobile clinics and supplementary immunisation activities (SIAs) could be incorporated in the pandemic response to avert any negative effects on utilisation of essential health services.

Declarations

This manuscript was submitted for publication with the permission of the Director KEMRI.

Funding

This research was funded in whole or in part by the Wellcome Trust Intermediate Fellow [Grant No. 201866]. For the purpose of Open Access, the author has applied a CC-BY public copyright licence to any author accepted manuscript version arising from this submission.

SW and **EAO** are supported through a Wellcome Trust Intermediate Fellow [Grant No.

For peer review only

201866]. **LM**,**JK, AN**, **GM**, **TT** and **CP** are supported through Funds from the Wellcome Trust [Grant No. 207522] awarded to Prof. Mike English (**ME**) as a senior Fellowship together with additional funds from a Wellcome Trust core grant awarded to the KEMRI-Wellcome Trust Research Programme [Grant No. 092654]. SW, LM, GM, TT, ME and EAO acknowledge the support of the Wellcome Trust to the Kenya Major Overseas Programme [Grant No. 203077].

Contributors

SW: Conceptualisation; Data curation; Formal analysis; Investigation; Methodology; Software; Validation; Visualisation; Writing - original draft. **LM**: Data curation; Formal analysis; Investigation; Methodology; Software; Validation; Visualisation; Writing - review & editing. **GM**: Data curation; Investigation; Software; Validation; Visualisation; Writing - review & editing. **AN**: Data curation; Formal analysis; Investigation; Software; Validation; Visualisation; Writing - review & editing. **JK**: Data curation; Formal analysis; Investigation; Software; Validation; Visualisation; Writing - review & editing. **TT**: Data curation; Investigation; Validation; Writing - review & editing **CP**: Data curation; Investigation; Validation; Writing - review & editing **ME**: Data curation; Investigation; Validation; Writing - review & editing. **EAO**: Data curation; Investigation; Validation; Funding acquisition; Writing - review & editing.

Data availability statement

Aggregated DHIS2 data is available online with access provided by Ministry

For peer review only

of Health https://hiskenya.org/dhis-web-commons/security/login.action.

Ethics approval and consent to participate

The study does not contain any individual person's data.

Consent for publication

Not applicable.

Competing interests

The authors declare that they have no competing interests

Patient and public involvement

We have used secondary aggregated routine health information data available online and did not directly engage patients.

Figure and table legend

Table 1: Description of indicators evaluated in this study.

Table 2: Segmented regression model estimates. Showing rate ratios (R.R.) alongside 95% confidence intervals (CI) and p-values.

Figure 1: Daily seven moving average trends of COVID-19 cases in Kenya showing various interventions initiated by the government.

Figure 2: Temporal trends in monthly immunisation and outpatient attendance nationally and by year

For peer review only

Figure 3: Statistical Process Control chart of immunisation, antenatal care and outpatient services. Horizontal dashed lines represent the 3-standard deviation mark.

Figure 4: Fitted lines of segmented regression models for outpatient and antenatal care attendance. Vertical lines represent the month (March 2020) COVID-19 was announced in Kenya and as a pandemic by the WHO.

Supplementary Files

Supplementary File 1: Number of health facilities analysed for each indicator including health facilities excluded for not reporting any month and those with less than 30% of months reported.

Supplementary File 2: Visual distribution patterns of missing data across all the health facilities analysed from DHIS 2

Supplementary File 3: Sensitivity analyses model estimates

For peer review only

References

1. John Hopkins University. COVID-19 Dashboard [Available from: https://coronavirus.jhu.edu/.

2. Sam SO, Pokhariyal GP, Rogo K et al. Otoi-NARIMA model for forecast seasonality of COVID-19 waves: Case of Kenya. 2021.

3. Uyoga S, Adetifa IM, Karanja HK et al. Seroprevalence of anti–SARS-CoV-2 IgG antibodies in Kenyan blood donors. *Science*. 2021;371(6524):79-82.

4. WHO. Kenya receives COVID-19 vaccines and launches landmark national campaign 2021 [01/07/2021]. Available from: https://www.afro.who.int/news/kenya-receives-covid-19-vaccines-and-launches-landmark-national-campaign.

5. Bai HM, Zaid A, Catrin S et al. The socio-economic implications of the coronavirus pandemic (COVID-19): A review. *Int J Surg*. 2020;8(4):8-17.

6. Quaife M, Van Zandvoort K, Gimma A et al. The impact of COVID-19 control measures on social contacts and transmission in Kenyan informal settlements. *BMC Med*. 2020;18(1):1-11.

7. Parpia AS, Ndeffo-Mbah ML, Wenzel NS et al. Effects of response to 2014–2015 Ebola outbreak on deaths from malaria, HIV/AIDS, and tuberculosis, West Africa. *Emerg Infect Dis*. 2016;22(3):433.

For peer review only

8. Riley T, Sully E, Ahmed Z et al. Estimates of the potential impact of the COVID-19 pandemic on sexual and reproductive health in low-and middle-income countries. *International Perspectives on Sexual and Reproductive Health*. 2020;46:73-6.

9. Roberton T, Carter ED, Chou VB et al. Early estimates of the indirect effects of the COVID-19 pandemic on maternal and child mortality in low-income and middle-income countries: a modelling study. *The Lancet Global Health*. 2020;8(7):e901-e8.

10. Barden-O'Fallon J, Barry MA, Brodish P et al. Rapid assessment of Ebola-related implications for reproductive, maternal, newborn and child health service delivery and utilization in Guinea. *PLoS currents*. 2015;7.

11. Bolkan HA, van Duinen A, Samai M et al. Admissions and surgery as indicators of hospital functions in Sierra Leone during the west-African Ebola outbreak. *BMC Health Serv Res*. 2018;18(1):846.

12. Brolin Ribacke KJ, van Duinen AJ, Nordenstedt H et al. The Impact of the West Africa Ebola Outbreak on Obstetric Health Care in Sierra Leone. *PLoS One*. 2016;11(2).

13. Elston J, Moosa A, Moses F et al. Impact of the Ebola outbreak on health systems and population health in Sierra Leone. *Journal of Public Health*. 2016;38(4):673-8.

14. Takahashi S, Metcalf CJE, Ferrari MJ et al. Reduced vaccination and the risk of measles and other childhood infections post-Ebola. *Science*. 2015;347(6227):1240-2.

For peer review only

15. Chang H-J, Huang N, Lee C-H et al. The impact of the SARS epidemic on the utilization of medical services: SARS and the fear of SARS. *Am J Public Health*. 2004;94(4):562-4.

16. Siedner MJ, Kraemer JD, Meyer MJ et al. Access to primary healthcare during lockdown measures for COVID-19 in rural South Africa: an interrupted time series analysis. *BMJ open*. 2020;10(10):e043763.

17. Barasa E, Kazungu J, Orangi S et al. Assessing the Indirect Health Effects of the COVID-19 Pandemic in Kenya. *Center for Global Development Forthcoming at https://www cgdev org/publication/assessing-indirect-health-effects-covid-19-pandemic-kenya*. 2021.

18. McQuaid C, Vassall A, Cohen T et al. The impact of COVID-19 on TB: a review of the data. *The International Journal of Tuberculosis and Lung Disease*. 2021;25(6):436-46.

19. Hategeka C, Carter SE, Chenge FM et al. Impact of the COVID-19 pandemic and response on the utilisation of health services during the first wave in Kinshasa, the Democratic Republic of the Congo. *medRxiv*. 2021.

20. Irimu G, Ogero M, Mbevi G et al. Tackling health professionals' strikes: an essential part of health system strengthening in Kenya. *BMJ global health*. 2018;3(6).

21. Kwak SK, Kim JH. Statistical data preparation: management of missing values and outliers. *Korean J Anesthesiol*. 2017;70(4):407.

For peer review only

22. WHO. Data quality review: a toolkit for facility data quality assessment. Module 2. Desk review of data quality 2017 [Available from: https://apps.who.int/iris/bitstream/handle/10665/259225/9789241512732-eng.pdf?sequence=1&isAllowed=y.

23. Rubin DB. Inference and missing data. *Biometrika*. 1976;63(3):581-92.

24. Rubin DB. Multiple imputation after 18+ years. *Journal of the American statistical Association*. 1996;91(434):473-89.

25. Quartagno M. Multiple Imputation for Individual Patient Data Meta-Analyses: London School of Hygiene & Tropical Medicine; 2016.

26. Quartagno M, Carpenter J, Quartagno MM et al. Package 'jomo'. 2020.

27. Quartagno M, Grund S, Carpenter J. Jomo: a flexible package for two-level joint modelling multiple imputation. *R Journal*. 2019;9(1).

28. Benneyan JC. The design, selection, and performance of statistical control charts for healthcare process improvement. *International Journal of Six Sigma and Competitive Advantage*. 2008;4(3):209-39.

29. Killick R, Eckley I. changepoint: An R package for changepoint analysis. *Journal of statistical software*. 2014;58(3):1-19.

30. Taylor WA. Change-point analysis: a powerful new tool for detecting changes. 2000.

For peer review only

31. Wagner AK, Soumerai SB, Zhang F et al. Segmented regression analysis of interrupted time series studies in medication use research. *J Clin Pharm Ther*. 2002;27(4):299-309.

32. Bernal JL, Cummins S, Gasparrini A. Interrupted time series regression for the evaluation of public health interventions: a tutorial. *Int J Epidemiol*. 2017;46(1):348-55.

33. Gardner W, Mulvey EP, Shaw EC. Regression analyses of counts and rates: Poisson, overdispersed Poisson, and negative binomial models. *Psychol Bull*. 1995;118(3):392.

34. Ver Hoef JM, Boveng PL. Quasi-Poisson vs. negative binomial regression: how should we model overdispersed count data? *Ecology*. 2007;88(11):2766-72.

35. Bozdogan H. Model selection and Akaike's information criterion (AIC): The general theory and its analytical extensions. *Psychometrika*. 1987;52(3):345-70.

36. Prais SJ, Winsten CB. Trend estimators and serial correlation. Cowles Commission discussion paper Chicago; 1954.

37. Nelson BK. Time series analysis using autoregressive integrated moving average (ARIMA) models. *Acad Emerg Med*. 1998;5(7):739-44.

38. Schaffer AL, Dobbins TA, Pearson S-A. Interrupted time series analysis using autoregressive integrated moving average (ARIMA) models: a guide for evaluating large-scale health interventions. *BMC Med Res Methodol*. 2021;21(1):1-12.

For peer review only

39. Bhaskaran K, Gasparrini A, Hajat S et al. Time series regression studies in environmental epidemiology. *Int J Epidemiol*. 2013;42(4):1187-95.

40. Rubin DB. Multiple imputation for nonresponse in surveys: John Wiley & Sons; 2004.

41. Masresha BG, Luce Jr R, Shibeshi ME et al. The performance of routine immunization in selected African countries during the first six months of the COVID-19 pandemic. *The Pan African Medical Journal*. 2020;37(Suppl 1).

42. SHIKUKU DN, Nyaoke I, Gichuru S et al. Early indirect impact of COVID-19 pandemic on utilization and outcomes of reproductive, maternal, newborn, child and adolescent health services in Kenya. *medRxiv*. 2020.

43. Burt JF, Ouma J, Amone A et al. Indirect Effects of COVID-19 on Maternal, Neonatal, Child, Sexual and Reproductive Health Services in Kampala, Uganda. *medRxiv*. 2021.

44. Dorward J, Khubone T, Gate K et al. The impact of the COVID-19 lockdown on HIV care in 65 South African primary care clinics: an interrupted time series analysis. *The Lancet HIV*. 2021;8(3):e158-e65.

45. Adelekan T, Mihretu B, Mapanga W et al. Early effects of the COVID-19 pandemic on family planning utilisation and termination of pregnancy services in Gauteng, South Africa: March–April 2020. *Wits Journal of Clinical Medicine*. 2020;2(2):145-52.

For peer review only

46. Wanyana D, Wong R, Hakizimana D. Rapid assessment on the utilization of maternal and child health services during COVID-19 in Rwanda. *Public Health Action*. 2021;11(1):12-21.

47. Chanchlani N, Buchanan F, Gill PJ. Addressing the indirect effects of COVID-19 on the health of children and young people. *CMAJ*. 2020;192(32):E921-E7.

48. Franchini S, Spessot M, Landoni G et al. Stranger months: how SARS-CoV-2, fear of contagion, and lockdown measures impacted attendance and clinical activity during February and March 2020 at an urban Emergency Department in Milan. *Disaster Med Public Health Prep*. 2020:1-10.

49. Migliori GB, Thong PM, Akkerman O et al. Worldwide effects of coronavirus disease pandemic on tuberculosis services, January–April 2020. *Emerg Infect Dis*. 2020;26(11):2709.

50. Murewanhema G, Makurumidze R. Essential health services delivery in Zimbabwe during the COVID-19 pandemic: perspectives and recommendations. *The Pan African Medical Journal*. 2020;35(Suppl 2).

51. Partnership for Evidence-Based Response to COVID-19 (PERC). Using data to find a balance,Special report series: Disruption to essential health services in Africa during COVID-19 2020 [cited 2021 21/06/2021]. Available from: https://preventepidemics.org/wp-content/uploads/2020/11/PERC-Brief-Essential-Services_Report_1120.pdf.

For peer review only

52. Edie GEHE, Obinchemti TE, Tamufor EN et al. Perceptions of antenatal care services by pregnant women attending government health centres in the Buea Health District, Cameroon: a cross sectional study. *Pan Afr Med J*. 2015;21(1).

53. Brown CA, Sohani SB, Khan K et al. Antenatal care and perinatal outcomes in Kwale district, Kenya. *BMC Pregnancy Childbirth*. 2008;8(1):1-11.

54. Shimels T, Asrat Kassu R, Bogale G et al. Magnitude and associated factors of poor medication adherence among diabetic and hypertensive patients visiting public health facilities in Ethiopia during the COVID-19 pandemic. *PLoS One*. 2021;16(4):e0249222.

55. Apicella M, Campopiano MC, Mantuano M et al. COVID-19 in people with diabetes: understanding the reasons for worse outcomes. *The lancet Diabetes & endocrinology*. 2020.

56. Agutu CA, Oduor TH, Kombo BK et al. High patient acceptability but low coverage of provider-initiated HIV testing among adult outpatients with symptoms of acute infectious illness in coastal Kenya. *PLoS One*. 2021;16(2):e0246444.

57. Mugo PM, Micheni M, Shangala J et al. Uptake and acceptability of oral HIV self-testing among community pharmacy clients in Kenya: a feasibility study. *PLoS One*. 2017;12(1):e0170868.

58. Aiello AE, Coulborn RM, Perez V et al. Effect of hand hygiene on infectious disease risk in the community setting: a meta-analysis. *Am J Public Health*. 2008;98(8):1372-81.

For peer review only

59. Liang M, Gao L, Cheng C et al. Efficacy of face mask in preventing respiratory virus transmission: A systematic review and meta-analysis. *Travel Med Infect Dis*. 2020;36:101751.

60. WHO. Analysing and using routine data to monitor the effects of COVID-19 on essential health services 2021 [01/06/2021]. Available from: https://www.who.int/bulletin/volumes/95/10/17-194399/en/.

61. Hagel C, Paton C, Mbevi G et al. Data for tracking SDGs: challenges in capturing neonatal data from hospitals in Kenya. *BMJ Global Health*. 2020;5(3):e002108.

62. Kihuba E, Gathara D, Mwinga S et al. Assessing the ability of health information systems in hospitals to support evidence-informed decisions in Kenya. *Global health action*. 2014;7(1):24859.

For peer review only

Table 1: Description of indicators and Ministry of Health (MOH) source forms used to capture the data

Category	Description	Assigned names in this study	Source form
Immunization	BCG vaccine doses administered	BCG	MOH 710
	Oral polio vaccine doses administered	OPV dose 1, dose 2 & dose 3	MOH 710
	Rotavirus vaccine doses administered	Rotavirus dose 1 & dose 2	MOH 710
	Pneumococcal conjugate vaccine doses administered	Pneumococcal dose 1, dose 2 & dose 3	MOH 710
	DPT vaccine doses administered	DPT 1, 2 & 3	MOH 710
	Inactivated polio vaccine doses administered	IPV	MOH 710
	Measles vaccine doses administered	Measles dose 1 & dose 2	MOH 710
Outpatient visits	Antenatal care first visit	ANC 1	MOH 711
	Antenatal care fourth visits	ANC 4	MOH 711
	Outpatient department visits in under-fives	OPD < 5 years	MOH 705A
	Outpatient department visits in over-fives	OPD > 5 years	MOH 705B
	Outpatient department visits with pneumonia in under-fives	OPD Pneumonia < 5 years	MOH 705 A
	Outpatient department visits with pneumonia in over-fives	OPD Pneumonia > 5 years	MOH 705B
	Number of new cases of diabetes	Diabetes new cases	MOH 705 A & B
	Number of new plus revisits of diabetes cases	Diabetes total cases	MOH 705 A & B
	Number of new hypertension cases	Hypertension new cases	MOH 705 A & B
	Number of new plus revisits of hypertension cases	Hypertension total cases	MOH 705 A & B
	Number of HIV tests performed	HIV tests performed	MOH 731

For peer review only

Table 2: Segmented regression model estimates. Showing rate ratios (RR) alongside 95% confidence intervals (CI) and p-values.

Covariate	OPD < 5 years			OPD > 5 years			OPD Pneumonia < 5 years			OPD Pneumonia > 5 years		
	RR	95%CI	P-value	RR	95%CI	P-value	RR	95%CI	P-value	RR	95%CI	P-value
COVID-19	0.50	(0.44-0.57)	<0.01	0.65	(0.57-0.75)	<0.01	0.43	(0.38-0.47)	<0.01	0.62	(0.55-0.70)	<0.01
Time	1.00	(0.99-1.01)	0.15	1.00	(1.00-1.01)	0.02	1.01	(1.00-1.01)	<0.01	1.00	(0.99-1.01)	0.05
Trend	1.05	(1.03-1.06)	<0.01	1.02	(1.00-1.04)	0.03	1.07	(1.05-1.08)	<0.01	1.03	(1.02-1.05)	<0.01
	ANC 1			ANC 4			Diabetes new cases			Diabetes total cases		
	RR	95%CI	P-value	RR	95%CI	P-value	RR	95%CI	P-value	RR	95%CI	P-value
COVID-19	0.96	(0.83-1.10)	0.55	0.86	(0.80-0.93)	<0.01	1.17	(0.89-1.52)	0.25	0.95	(0.93-0.97)	<0.01
Time	1.00	(0.99-1.00)	0.61	1.00	(0.99-1.00)	0.13	0.99	(0.98-1.00)	0.13	1.00	(1.00-1.01)	<0.01
Trend	1.01	(0.99-1.03)	0.12	1.00	(0.99-1.01)	0.90	0.99	(0.97-1.01)	0.57	1.00	(1.00-1.00)	0.05
	Hypertension new cases			Hypertension total cases			HIV Tests Performed					
	RR	95%CI	P-value	RR	95%CI	P-value	RR	95%CI	P-value			

For peer review only

**COVID-19**	0.87	(0.75-1.00)	0.05	0.89	(0.82-0.96)	<0.01	0.97	(0.94-0.99)	0.01	
**Time**	1.00	(0.99-1.01)	0.81	1.01	(1.00-1.01)	<0.01	0.97	(0.97-0.97)	<0.01	
**Trend**	1.00	(0.99-1.01)	0.59	1.00	(0.99-1.01)	0.90	1.01	(1.00-1.01)	<0.01	

For peer review only

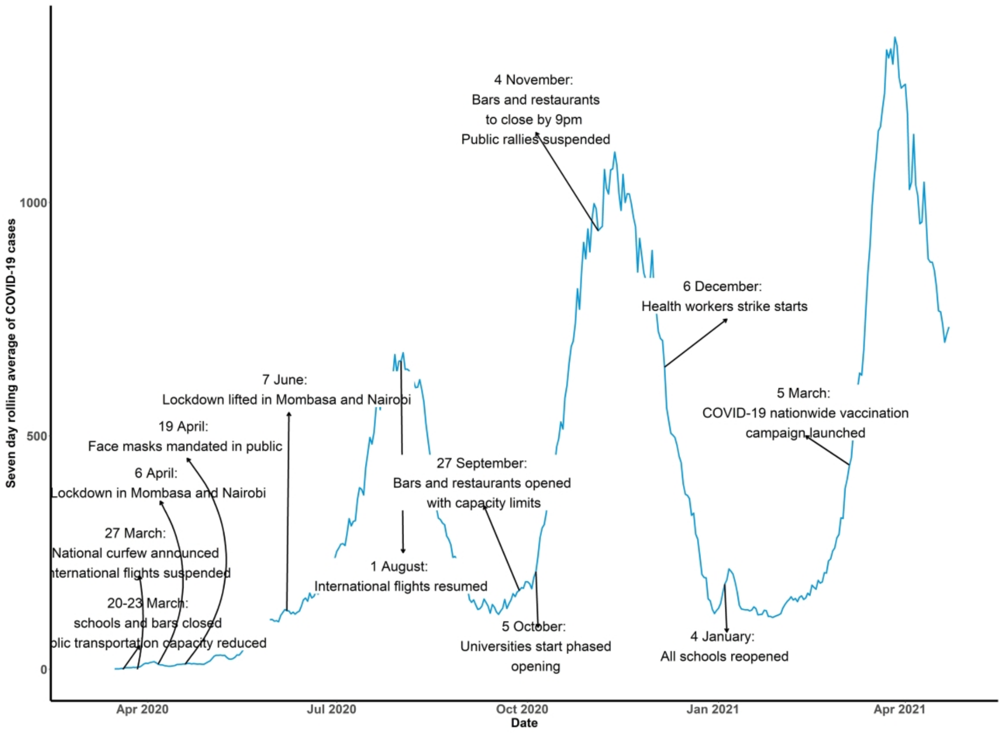

Daily seven moving average trends of COVID-19 cases in Kenya showing various interventions initiated by the government.

190x142mm (500 x 500 DPI)

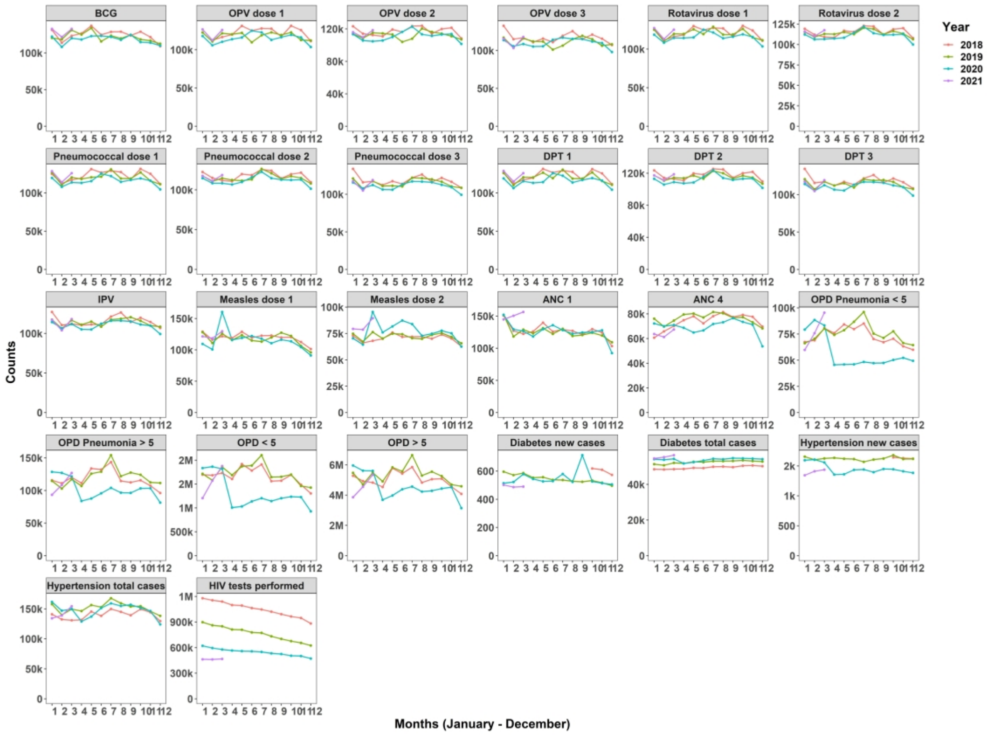

Temporal trends in monthly immunisation and outpatient attendance nationally and by year

190x142mm (500 x 500 DPI)

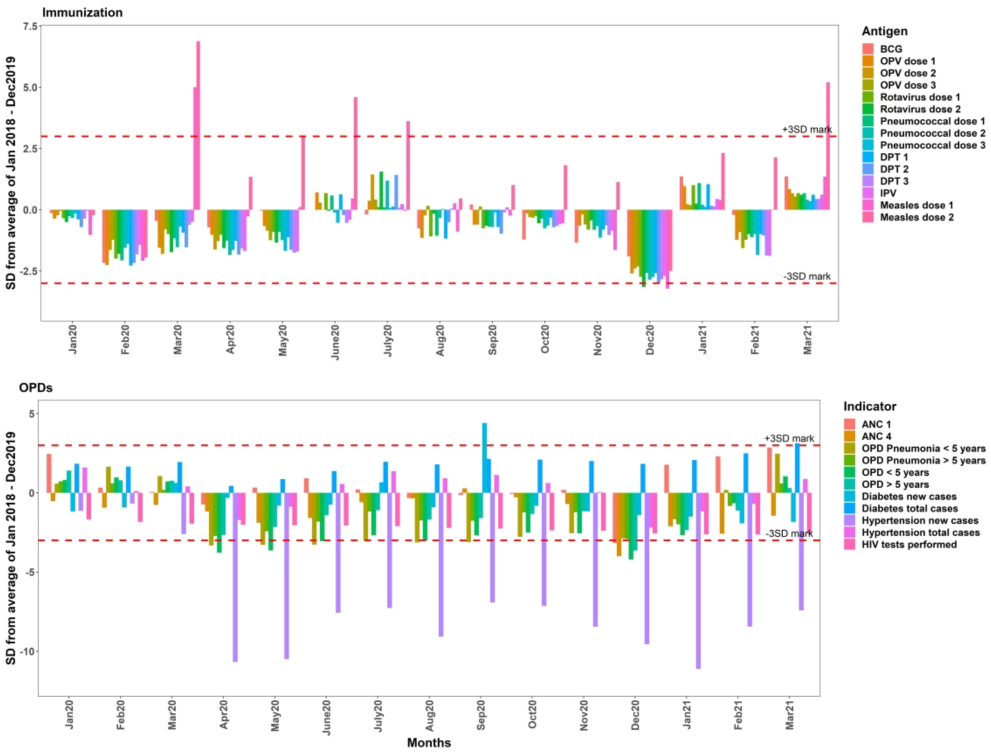

Statistical Process Control chart of immunisation, antenatal care and outpatient services. Horizontal dashed lines represent the 3-standard deviation mark.

190x142mm (500 x 500 DPI)

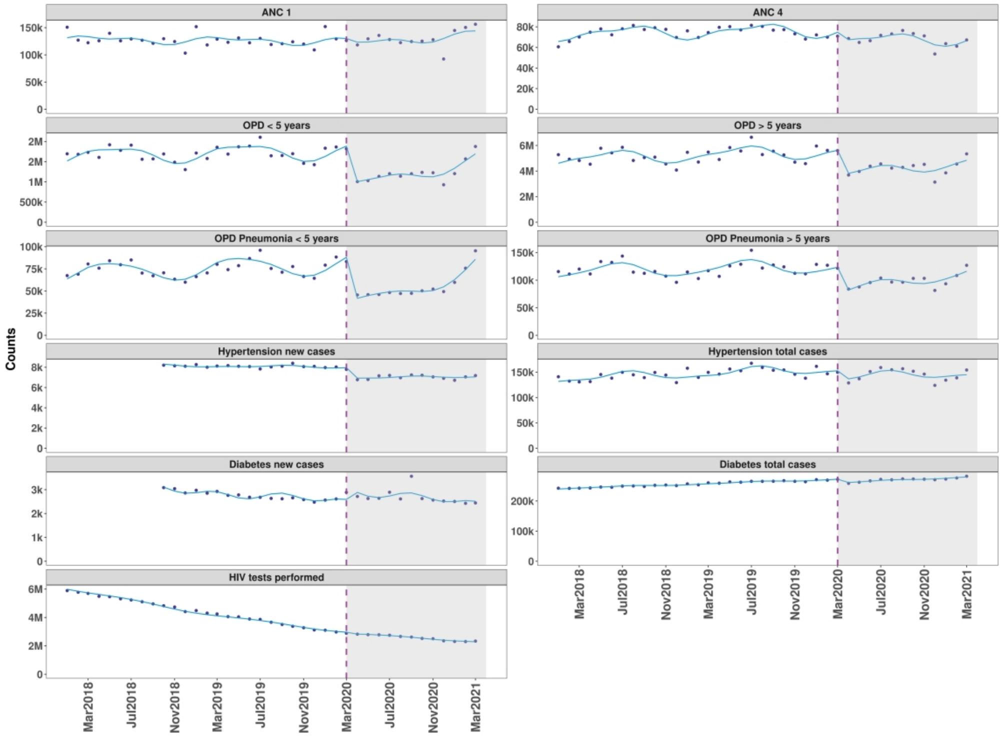

Fitted lines of segmented regression models for outpatient and antenatal care attendance. Vertical lines represent the month (March 2020) COVID-19 was announced in Kenya and as a pandemic by the WHO.

190x142mm (500 x 500 DPI)

SI Table 1: Number and percentage of health facilities analysed for each indicator. It shows number of facilities that did not report any month and those that were imputed (health facilities with more than 30% of months reported)

Indicator	All hospitals expected to report in DHIS2	Number of health facilities imputed	Number of health facilities with no reported data	Percent of health facilities analysed out of those reporting at least a month
BCG	8063	6509	352	84
DPT1	8063	7142	130	90
DPT2	8063	7141	140	90
DPT3	8063	7136	124	90
IPV	8063	7089	144	90
Measles1	8063	7166	125	90
Measles2	8063	6578	230	84
OPV1	8063	7134	128	90
OPV2	8063	7144	140	90
OPV3	8063	7124	123	90
Pneum1	8063	7139	132	90
Pneum2	8063	7143	141	90
Pneum3	8063	7145	129	90
Rota1	8063	7126	130	90
Rota2	8063	7114	146	90
ANC 1	13595	7768	4450	85
ANC 4	13595	7768	4450	85
OPD > 5 Female	13595	9434	3156	90
OPD > 5 Male	13595	9431	3153	90
OPD < 5 Female	13595	9246	3274	90
OPD < 5 Male	13595	9250	3276	90
OPD Pneumonia > 5	13595	7933	3264	77
OPD Pneumonia < 5	13798	6976	3784	70
Diabetes new cases	13752	72	13472	26
Diabetes total cases	13752	4220	4914	48
Hypertension new cases	13752	121	13454	41
Hypertension total cases	13757	7381	3765	74
HIV tests performed	13752	6789	5674	84

For peer review only

SI Figure 1: Missing data patterns plot for immunization indicators showing number of reported months by health facilities.

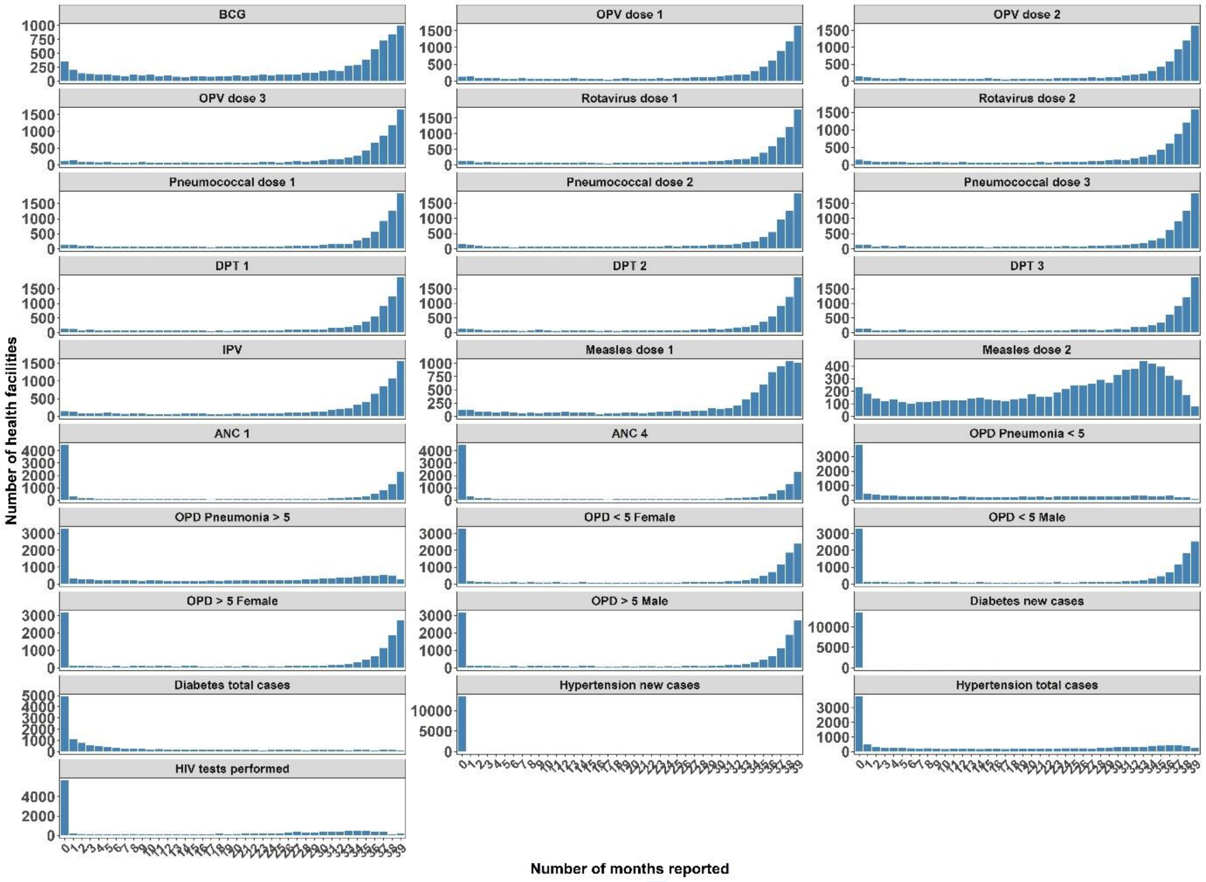

The x – axis shows the number of months reported by health facilities (0 to 39). 0 to the left means the health facilities did not report any month or may not be offering the service, while 39 means the health facilities reported all months.

SI Figure 1: Multiple change point analysis plots showing significant shifts in attendance

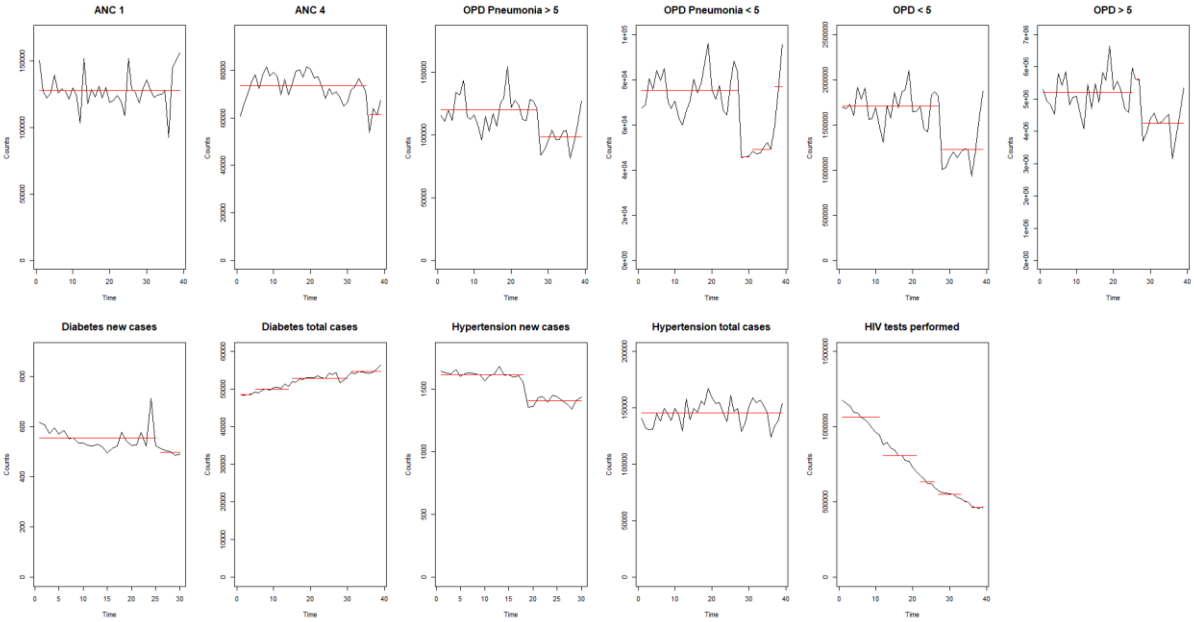

For peer review only

SI Table 1: Interrupted time series models comparing estimates before and after excluding strike period

		OPD < 5			OPD > 5			OPD Pneumonia < 5			OPD Pneumonia > 5		
**Ownership**		**RR**	**95%CI**	**P-value**	**RR**	**95%CI**	**P-value**	**RR**	**95%CI**	**P-value**	**RR**	**95%CI**	**P-value**
**Primary**	**COVID-19**	0.50	(0.44-0.57)	<0.01	0.65	(0.57-0.75)	<0.01	0.43	(0.38-0.47)	<0.01	0.62	(0.55-0.70)	<0.01
	**Time**	1.00	(0.99-1.01)	0.15	1.00	(1.00-1.01)	0.02	1.01	(1.00-1.01)	<0.01	1.00	(0.99-1.01)	0.05
	**Trend**	1.05	(1.03-1.06)	<0.01	1.02	(1.00-1.04)	0.03	1.07	(1.05-1.08)	<0.01	1.03	(1.02-1.05)	<0.01
**Excluding Strike**	**COVID-19**	0.45	(0.39-0.52)	<0.01	0.60	(0.53-0.68)	<0.01	0.39	(0.33-0.47)	<0.01	0.58	(0.52-0.66)	<0.01
	**Time**	1.00	(1.00-1.01)	0.13	1.01	(1.00-1.01)	<0.01	1.01	(1.00-1.01)	0.02	1.00	(1.00-1.01)	0.03
	**Trend**	1.09	(1.06-1.11)	<0.01	1.05	(1.03-1.07)	<0.01	1.10	(1.07-1.13)	<0.01	1.06	(1.04-1.08)	<0.01
		**ANC 1**			**ANC 4**			**Diabetes new cases**			**Diabetes total cases**		
		**RR**	**95%CI**	**P-value**	**RR**	**95%CI**	**P-value**	**RR**	**95%CI**	**P-value**	**RR**	**95%CI**	**P-value**
**Primary**	**COVID-19**	0.96	(0.83-1.10)	0.55	0.86	(0.80-0.93)	<0.01	1.17	(0.89-1.52)	0.25	0.95	(0.93-0.97)	<0.01
	**Time**	1.00	(0.99-1.00)	0.61	1.00	(0.99-1.00)	0.13	0.99	(0.98-1.00)	0.13	1.00	(1.00-1.01)	<0.01
	**Trend**	1.01	(0.99-1.03)	0.12	1.00	(0.99-1.01)	0.90	0.99	(0.97-1.01)	0.57	1.00	(1.00-1.00)	0.05
**Excluding Strike**	**COVID-19**	0.96	(0.84-1.09)	0.52	0.83	(0.77-0.89)	<0.01	1.12	(0.85-1.48)	0.43	0.94	(0.92-0.96)	<0.01
	**Time**	1.00	(1.00-1.00)	0.44	1.00	(1.00-1.00)	0.05	0.99	(0.98-1.00)	0.12	1.00	(1.00-1.01)	<0.01
	**Trend**	1.02	(1.00-1.04)	0.06	1.01	(1.00-1.03)	0.02	1.01	(0.98-1.04)	0.73	1.01	(1.00-1.01)	<0.01
		**Hypertension new cases**			**Hypertension total cases**			**HIV Tests Performed**					
		**RR**	**95%CI**	**P-value**	**RR**	**95%CI**	**P-value**	**RR**	**95%CI**	**P-value**			
**Primary**	**COVID-19**	0.87	(0.75-1.00)	0.05	0.89	(0.82-0.96)	<0.01	0.97	(0.94-0.99)	0.01			
	**Time**	1.00	(0.99-1.01)	0.81	1.01	(1.00-1.01)	<0.01	0.97	(0.97-0.97)	<0.01			
	**Trend**	1.00	(0.99-1.01)	0.59	1.00	(0.99-1.01)	0.90	1.01	(1.01-1.01)	<0.01			
**Excluding Strike**	**COVID-19**	0.86	(0.74-1.00)	0.06	0.85	(0.79-0.92)	<0.01	0.97	(0.94-1.01)	0.11			
	**Time**	1.00	(0.99-1.01)	0.81	1.01	(1.00-1.01)	<0.01	0.97	(0.97-0.97)	<0.01			
	**Trend**	1.01	(0.99-1.02)	0.48	1.01	(1.00-1.03)	0.02	1.01	(1.00-1.01)	<0.01			

For peer review only

SI Table 2: Generalised estimating equations (GEE) results at health facility level showing rate ratios (RR) for COVID-19 intervention, time and trend alongside 95% confidence intervals for all indicators

		OPD < 5			OPD > 5			OPD Pneumonia < 5			OPD Pneumonia > 5		
Ownership		RR	95%CI	P-value	RR	95%CI	P-value	RR	95%CI	P-value	RR	95%CI	P-value
Primary	COVID-19	0.50	(0.44-0.57)	<0.01	0.65	(0.57-0.75)	<0.01	0.43	(0.38-0.47)	<0.01	0.62	(0.55-0.70)	<0.01
	Time	1.00	(0.99-1.01)	0.15	1.00	(1.00-1.01)	0.02	1.01	(1.00-1.01)	<0.01	1.00	(0.99-1.01)	0.05
	Trend	1.05	(1.03-1.06)	<0.01	1.02	(1.00-1.04)	0.03	1.07	(1.05-1.08)	<0.01	1.03	(1.02-1.05)	<0.01
GEE	COVID-19	0.50	(0.48-0.51)	<0.01	0.65	(0.64-0.66)	<0.01	0.42	(0.40-0.43)	<0.01	0.62	(0.60-0.64)	<0.01
	Time	1.00	(1.00-1.01)	<0.01	1.01	(1.00-1.01)	<0.01	1.01	(1.00-1.01)	<0.01	1.00	(1.00-1.01)	<0.01
	Trend	1.05	(1.04-1.05)	<0.01	1.02	(1.01-1.02)	<0.01	1.07	(1.06-1.07)	<0.01	1.03	(1.03-1.04)	<0.01
		ANC 1			ANC 4			Diabetes new cases			Diabetes total cases		
		RR	95%CI	P-value	RR	95%CI	P-value	RR	95%CI	P-value	RR	95%CI	P-value
Primary	COVID-19	0.96	(0.83-1.10)	0.55	0.86	(0.80-0.93)	<0.01	1.17	(0.89-1.52)	0.25	0.95	(0.93-0.97)	<0.01
	Time	1.00	(0.99-1.00)	0.61	1.00	(0.99-1.00)	0.13	0.99	(0.98-1.00)	0.13	1.00	(1.00-1.01)	<0.01
	Trend	1.01	(0.99-1.03)	0.12	1.00	(0.99-1.01)	0.90	0.99	(0.97-1.01)	0.57	1.00	(1.00-1.00)	0.05
GEE	COVID-19	0.96	(0.92-0.99)	0.01	0.87	(0.83-0.90)	<0.01	1.17	(0.89-1.53)	0.26	0.95	(0.93-0.98)	<0.01
	Time	1.00	(0.99-1.00)	0.05	1.00	(1.00-1.01)	0.01	0.99	(0.98-1.00)	0.13	1.00	(1.00-1.01)	<0.01
	Trend	1.01	(1.01-1.02)	<0.01	1.00	(0.99-1.01)	0.71	0.99	(0.98-1.01)	0.58	1.00	(1.00-1.00)	0.26
		Hypertension new cases			Hypertension total cases			HIV Tests Performed					
		RR	95%CI	P-value	RR	95%CI	P-value	RR	95%CI	P-value			
Primary	COVID-19	0.87	(0.75-1.00)	0.05	0.89	(0.82-0.96)	<0.01	0.97	(0.94-0.99)	0.01			
	Time	1.00	(0.99-1.01)	0.81	1.01	(1.00-1.01)	<0.01	0.97	(0.97-0.97)	<0.01			
	Trend	1.00	(0.99-1.01)	0.59	1.00	(0.99-1.01)	0.90	1.01	(1.01-1.01)	<0.01			
GEE	COVID-19	0.87	(0.74-1.02)	0.09	0.89	(0.85-0.92)	<0.01	0.96	(0.95-0.98)	<0.01			
	Time	1.00	(0.99-1.01)	0.83	1.01	(1.00-1.01)	<0.01	0.97	(0.97-0.97)	<0.01			
	Trend	1.00	(0.99-1.02)	0.70	1.00	(1.00-1.01)	0.83	1.01	(1.01-1.01)	<0.01			

For peer review only

BMJ Open

Quantifying the indirect impact of COVID-19 pandemic on utilisation of outpatient and immunisation services in Kenya: A longitudinal study using interrupted time series analysis

Journal:	*BMJ Open*
Manuscript ID	bmjopen-2021-055815.R1
Article Type:	Original research
Date Submitted by the Author:	07-Dec-2021
Complete List of Authors:	Wambua, Steven; KEMRI-Wellcome Trust Research Programme Nairobi, Population Health Unit<br>Malla, Lucas; KEMRI-Wellcome Trust Research Programme Nairobi, Health Services Unit<br>Mbevi, George; KEMRI-Wellcome Trust Research Programme Nairobi, Health Services Unit<br>Kandiah, Joel; University of Warwick, Mathematics Institute<br>Nwosu, Amen-Patrick; Oxford Centre for Global Health Research, Nuffield Department of Clinical Medicine<br>Tuti, Timothy; KEMRI-Wellcome Trust Research Programme Nairobi, Health Services Unit<br>Paton, Chris; Oxford Centre for Global Health Research, Nuffield Department of Clinical Medicine<br>Wambu, Bernard; Kenya Ministry of Health<br>English, Mike; KEMRI-Wellcome Trust Research Programme Nairobi, Health Services Unit; Oxford Centre for Global Health Research, Nuffield Department of Clinical Medicine<br>Okiro, Emelda ; KEMRI-Wellcome Trust Research Programme Nairobi, Population Health Unit; Oxford Centre for Global Health Research, Nuffield Department of Clinical Medicine
**Primary Subject Heading**:	Health services research
Secondary Subject Heading:	Public health, Global health
Keywords:	Public health < INFECTIOUS DISEASES, COVID-19, Paediatric infectious disease & immunisation < PAEDIATRICS, International health services < HEALTH SERVICES ADMINISTRATION & MANAGEMENT

SCHOLARONE™
Manuscripts

I, the Submitting Author has the right to grant and does grant on behalf of all authors of the Work (as defined in the below author licence), an exclusive licence and/or a non-exclusive licence for contributions from authors who are: i) UK Crown employees; ii) where BMJ has agreed a CC-BY licence shall apply, and/or iii) in accordance with the terms applicable for US Federal Government officers or employees acting as part of their official duties; on a worldwide, perpetual, irrevocable, royalty-free basis to BMJ Publishing Group Ltd ("BMJ") its licensees and where the relevant Journal is co-owned by BMJ to the co-owners of the Journal, to publish the Work in this journal and any other BMJ products and to exploit all rights, as set out in our licence.

The Submitting Author accepts and understands that any supply made under these terms is made by BMJ to the Submitting Author unless you are acting as an employee on behalf of your employer or a postgraduate student of an affiliated institution which is paying any applicable article publishing charge ("APC") for Open Access articles. Where the Submitting Author wishes to make the Work available on an Open Access basis (and intends to pay the relevant APC), the terms of reuse of such Open Access shall be governed by a Creative Commons licence – details of these licences and which Creative Commons licence will apply to this Work are set out in our licence referred to above.

Other than as permitted in any relevant BMJ Author's Self Archiving Policies, I confirm this Work has not been accepted for publication elsewhere, is not being considered for publication elsewhere and does not duplicate material already published. I confirm all authors consent to publication of this Work and authorise the granting of this licence.

For peer review only

Quantifying the indirect impact of COVID-19 pandemic on utilisation of outpatient and immunisation services in Kenya: A longitudinal study using interrupted time series analysis

Steven Wambua[1]*, Lucas Malla[2], George Mbevi[2], Joel Kandiah[4], Amen-Patrick Nwosu[3], Timothy Tuti[2], Chris Paton[3], Bernard Wambu[5], Mike English[2,3], Emelda A. Okiro[1,3].

[1]Population Health Unit, Kenya Medical Research Institute-Wellcome Trust Research Programme, Nairobi, Kenya

[2]Health Services Unit, KEMRI-Wellcome Trust Research Programme, Nairobi, Kenya

[3]Oxford Centre for Global Health Research, Nuffield Department of Clinical Medicine, University of Oxford, Oxford, U.K.

[4]University of Warwick, Mathematics Institute, Coventry, U.K

[5]Ministry of Health, Nairobi, Kenya

*Corresponding author

SW*:swambua@kemri-wellcome.org, PO BOX 43640-00100, Nairobi Kenya, Tel: 0718618560

Word Count: 4472

Abstract

Objective: In this study we assess the indirect impact of COVID-19 on utilization of immunisation and outpatient services in Kenya.

For peer review only

Design: Longitudinal study

Setting: Data were analysed from all healthcare facilities reporting to Kenya's health information system (KHIS) from January 2018 to March 2021. Multiple imputation was used to address missing data, interrupted timeseries analysis was used to quantify the changes in utilization of services and sensitivity analysis was carried out to assess robustness of estimates.

Exposure of interest: COVID-19 outbreak and associated interventions

Outcome measures: Monthly attendance to health facilities. We assessed changes in immunization and various outpatient services nationally.

Results

Before the first case of COVID-19 and pursuant intervention measures in March 2020, uptake of health services was consistent with historical levels. There was significant drops in attendance (level changes) in April 2020 for overall outpatient visits for under-fives (RR=0.50 95% CI (0.44-0.57)), under-fives with pneumonia (RR=0.43 95% CI (0.38-0.47)), overall over-five visits (RR=0.65 95% CI (0.57-0.75), over-fives with pneumonia (RR=0.62 95% CI (0.55-0.70)), fourth antenatal care visit (RR=0.86 95% CI (0.80-0.93)), total hypertension (RR=0.89 95% CI (0.82-0.96)), diabetes cases (RR=0.95 95% CI (0.93-0.97)) and HIV testing (RR=0.97 95% CI (0.94-0.99)). Immunization services, first antenatal care visits, new cases of hypertension and diabetes were not affected. The post-COVID-19 trend was increasing, with more recent data suggesting reversal of effects and health services reverting to expected levels as of March 2021.

Conclusion

COVID-19 pandemic has had varied indirect effects on utilization of health services in Kenya. There is need for proactive and targeted interventions to reverse these effects as part of the pandemic's response to avert non-COVID-19 indirect mortality.

Keywords: COVID-19, SARS-CoV-2, outpatient services, immunization, DHIS2

For peer review only

Strengths and limitations of this study

- This analysis is strengthened by use of a broad set of health services indicators and over a large number of health facilities nationally and a longer time period (39 months) allowing for the adjustment of pre-COVID-19 trends.
- We have adjusted for factors such as health workers strikes and missing data in the analysis strengthening the validity of the results.
- Data was analysed across the whole health care system in Kenya (both public and private sector) therefore can be used to predict impact in other similar settings.
- COVID-19 outbreak and associated public health measures were not random. Other concurrent unmeasured factors or shocks could have contributed, however small, to the changes.
- This study doesn't allow for in-depth evaluation of the specific causes of the trends observed within a qualitative framework because it was purely quantitative.

For peer review only

Introduction

The novel coronavirus (COVID-19) outbreak was declared a global pandemic by the World Health Organization (WHO) on March 11, 2020. By 6th May 2021, 156 million cases and 3.2 million deaths have been reported globally,(1). Since the first case of COVID-19 was reported in Kenya on 13th March 2020, 162,098 cases and 2850 deaths were reported by 6th May 2021,(1). The government, in attempt to control the spread of the pandemic, instituted a raft of interventions. Consequently, beyond the pandemic's direct impact on the population health, indirect effects due to the control measures, changes

in public and clinician behaviour and health system reorganization are likely to manifest in changes to utilisation of essential health services.

The country has experienced three waves of the pandemic,(2). The first wave peaked in July/August 2020,(3) and in March 2021, the country experienced the third wave with the highest daily cases recorded since the start of pandemic. Throughout this period, a series of public health measures have been instituted by government authorities such as restrictions in movement, international travel and suspension of gatherings in various public places. In March 5th 2021, the COVID-19 vaccination campaign targeting 1.02 million health workers and those above the age of 58 years was launched,(4).

The public health interventions are expected to have economic and social impacts such as reductions in manufacturing, access to employment and basic necessities,(5, 6). Consequently, access and utilisation of essential health services are likely to be affected,(7). Early modelled predictions showed reductions in utilization of health services,(8, 9). In addition, studies during previous epidemics in sub-Saharan Africa reported a reduction in utilisation of essential health services during and after outbreaks,(10-14). Various population groups are likely to be affected differently, with children and women at a higher risk,(10, 15). These interruptions in health service utilisation are raising concerns of increased morbidity and mortality for non-COVID-19 illnesses and especially for childcare services,(9). Although recent studies have reported variable impact of the pandemic on various health services, the impact on administration of vaccines and monitoring a broad set of essential services over a longer -observation period after the pandemic was announced by WHO has not been evaluated rigorously in Kenya,(16-19).

Using the Kenya's routine health information system implemented through the District Health Information Software version 2 (DHIS 2), a database where all health facilities in Kenya are expected to report services they offered in a given month, this study aimed to assess the indirect impact of COVID-19 on utilisation of varied basic essential health services nationally.

Methods

Timeline of events

For peer review only

Pre-COVID-19 measures

Two months before the first case of COVID-19 was reported in Kenya, the government increased preparedness towards the pandemic. The preparedness measures included monitoring suspected cases of COVID-19 at points of entry to the country, increasing capacity for testing and isolation centres, providing healthcare workers with information and tools for dealing with COVID-19 cases and enactment of an emergency response committee (20, 21).

Post-COVID-19 control measures

Control measures to manage COVID-19 spread were first enacted on 13th March 2020 (22). These were suspension of public gatherings including places of worship and limiting the number of people attending weddings and funerals. Institution of learning, bars and restaurants were also closed. Travel restrictions into and out of the country were put in place and the national dusk-to-dawn curfew was introduced. A month later, restrictions in movement into and out of counties with highest cases of COVID-19 were instituted and restaurants resumed operations under strict guidelines of social and physical distancing, temperature checks when accessing the restaurants and handwashing. In the month of May 2020, the government ceased movement into and out of the country through two neighbouring countries (Tanzania and Somalia). Home-based care was introduced for patients with COVID-19 in June 2020 and in July 2020 the government started relaxing restrictions on movement and local air travel and phased re-opening of churches and other places of worship. In August 2020, international air travel resumed and in September 2020, operation of bars resumed. This was followed by phased re-opening of schools and lifting of suspension on political gatherings in October 2020 and November 2020 respectively. Between December 2020 to February 2021 there was a national health workers strike triggered by demands for better working conditions such as provision of adequate Personal Protective Equipment (PPE), enhanced risk allowances and a health insurance cover. Although the length of the strike varied by health facilities and cadre of health workers, we couldn't obtain a database which tracks strikes nationally, and we therefore assumed most of the health facilities were on strike during the whole

For peer review only

period. All schools re-opened in January 2021. The timeline of COVID-19 control measures is presented in Figure 1.

------------------------------------Figure 1---

Data

Data Sources

District Health Information Software version 2 (DHIS2)

DHIS2 is an open-source software platform for data reporting by all the health facilities in a country. The primary goals of the system were to establish a centralized database with reporting capabilities at health facilities, to define and determine the standards for local and national health service reports and to connect service delivery and other health system input databases (23). Monthly aggregated hospital level data can be entered into the system using a variety of tools, including desktop computers, laptops, tablets and smartphones by health records and information officers (HRIOs) situated in various hospitals. For health facilities without a HRIO, data is sent to a central administrative unit where the data is aggregated and entered into the system. Strong technical capabilities, flexibility, cost-effectiveness, increased satisfaction and networking among stakeholders have been some of the strengths of DHIS2 reported in 11 countries (24).

Extracted data

We extracted monthly data from DHIS 2 for the period January 2018 to March 2021 on total outpatient visits (under and over-fives), the number of hypertension and diabetes cases and HIV tests performed, doses of immunisation antigens administered and antenatal care visits (the first (ANC 1) and fourth (ANC 4) visits). ANC 1 and ANC 4 are recommended by WHO as tracker indicators for antenatal care coverage and hence are reported in DHIS 2. A description of the indicators is presented in Table 1.

---Table 1--

For peer review only

Data were not available for the period January 2018 to September 2018 for hypertension and diabetes new cases. For both indicators and for relevant periods data were excluded from the analysis. We chose 2018 as a starting point because of prolonged health care worker strikes in 2017 which affected health services provision,(25) and consequently reporting. Data were cleaned to remove duplicated health facilities and those indicated as closed. Extreme outliers, defined as values that are more than 3 standard deviations from the mean of reported values for a given health facility,(26, 27), were identified, investigated and treated as missing. For each health facility, we obtained the administrative units, level of the facility (Level 2: Dispensaries with outpatient services only, Level 3: Comprehensive primary health care facilities, Level 4: primary referral hospitals, Level 5: Secondary referral hospitals and Level 6: national teaching and referral hospitals) and whether the health facility is private or public.

Statistical analysis

Missing data in DHIS 2

Missing data occurred for the indicators in a given month for a given health facility. Missingness varies by health facility and consistency in reporting overtime. Incompleteness in reports has been attributed to inadequate human resources, frequent power outages and slow internet connectivity, use of manual and electronic systems concurrently and frequent changes in DHIS 2 versions (28). Strategies to improve reporting such as improving clinical care documentation, motivation among staff, government commitment and extensive donor support have been identified as strategies to improve completeness in DHIS 2 (29, 30).

Handling missing data

To adjust for incompleteness in reporting, multiple imputation (MI) was performed,(31-33). MI has been shown to perform better in handling missing data in comparison to other methods(34). Missing monthly values were imputed using a mixed effects model in a joint modelling framework,(35, 36). Health facility ownership (public or private), level of health facility, time (month and year) and COVID-19 binary indicator (0 – months before pandemic and 1 – months post pandemic) were used as covariates

For peer review only

with the health facility as a clustering variable. MI was performed for health facilities with more than 30% of months reported (at least 12 months reported) to reduce uncertainty in imputed values and ensure generalizability of the estimates. The missing patterns for each indicator are presented in Additional File 1 SI Figure 1. The MI model specification has been provided in Additional File 2. Additionally, through a simulation study we found MI performance and efficiency was best when imputing for health facilities with more than 30% of months reported. The number of health facilities analysed is presented in Additional File 3 SI Table 1.

Interrupted time series analysis

Exploratory analyses

Data were aggregated monthly for all health facilities. Trends were plotted to visualise changes in utilisation of health services. Statistical process control (SPC) charts with the 2018-2019 average as a baseline were used to identify significant shifts in monthly values for 2020-2021. Values that are more than 3 standard deviations from the mean are considered significant shifts and were carried forward for interrupted time series analysis (37). Multiple change point analysis was applied to assess the influence of health worker strike on provision of health services,(38, 39).

Segmented regression

We conducted interrupted time series analyses using monthly attendance counts for each indicator as outcomes. The period running from January 2018 through March 2020 when the first case was identified was defined as pre-COVID-19 and April 2020 to March 2021 as post-COVID-19. For indicators where changes were observed in SPC analysis, segmented regression were performed to model attendance before and after COVID-19 was reported,(40, 41). The following equation specifies the model,(40);

$$\log(Y_t) = \beta_0 + \beta_1 * time_t + \beta_2 * COVID19_t + \beta_3 * time\ after\ COVID19_t$$

Where, Y_t is the attendance in month t; $time$ is a continuous indicator of time in months from January 2018; $COVID19$ is an indicator of time t occurring before ($COVID19 = 0$) or after ($COVID19 = 1$) the

For peer review only

outbreak, which was implemented at April 2021 in the series; and $time\ after\ COVID19$ is a continuous variable of the number of months after COVID-19 at time t. In the model, β_0 estimates the baseline level of attendance at time zero; β_1 estimates the change in monthly number of visits before COVID-19 (pre-existing trend); β_2 estimates the level change immediately after COVID-19 outbreak; β_3 estimates the change in the trend after COVID-19, compared with the pre-existing trend. A change in intercept (immediate COVID-19 effect) and change in slope (gradual COVID-19 effect) were hypothesised,(41).

A generalised linear model was applied assuming a negative binomial distribution. The negative binomial model was selected due to variations in attendance at health facility level. The intraclass correlation coefficients for each indicator are provided in Additional File 4 Table 1. We fitted two negative binomial models to account for over-dispersion, one without accounting for seasonality and another accounting for seasonality,(41-43). Model performance was evaluated using the Akaike's information criterion,(44). Model checking was conducted for autocorrelation using the Durbin-Watson statistic and autoregressive moving average (ARMA) models were fitted for indicators with serial autocorrelation,(45-47). The ARMA model fitted is presented below;

$$X_t = c + \varepsilon_t + \sum_{j=1}^{p} \varphi_i X_{t-i} + \sum_{j=1}^{q} \theta_i \varepsilon_{t-i}$$

Where φ is the AR model parameters, θ is the MA model parameters, c a constant and ε is the error term. We fitted the ARMA model using various combinations of p and q and selected the model with the lowest Arkeike Information Criteria (AIC). The *gcmr* package was used to implement the ARMA models (48). Seasonality was adjusted using Fourier terms by specifying the sine and cosine pairs as 2 and the length of the period as 12 as recommended by Bernal et al ,(49). Results were pooled across the multiple imputed datasets using Rubin's rules,(50). The negative binomial model, which was adjusted for seasonality was the best fitting model and its results are presented in this study. AIC values and the estimates from the negative binomial model where seasonality was not accounted for are provided in Additional File 4 Table 2 and Table 3.

For peer review only

As a form of sensitivity analysis, we fitted models excluding months when the national strike occurred and compared estimates with those where data included the strike. We also fitted health-facility level generalised estimating equations to test the impact of varying model assumptions on the primary model estimates and hence evaluate robustness of our results,(41).

Statistical significance was defined as p-values < 0.05. All analyses were performed using R (version 3.6.3).

Patient and public involvement

No patients were involved in this study. We have used secondary aggregated routine health information data available online.

Results

COVID-19 impact

-------------------------------------Figure 2---

Annual trends show the first antenatal care visits remained unaffected while the fourth visits experienced a downward trend from March 2020. Immunization services remained unaffected with observed spikes in administration of measles vaccines in March 2020. Utilization of outpatient services (overall and due to pneumonia) by under-fives experienced drops after March 2020. Reductions were also experienced in over-fives attendance, hypertension cases and diabetes attendance. HIV testing experienced a gradual decline over the years (Figure 2).

-------------------------------------Figure 3---

Further, SPC charts confirmed significant reductions (less than 3SD) in ANC 4 starting April 2020. Immunization services remained unaffected during the same period, with significant increase (more than 3SD) in measles vaccination in March 2020. Moreover, significant reductions in under-fives attendance, over-fives attendance and new visits by hypertensive patients were observed starting April 2020 with no significant reductions for HIV testing and diabetes visits (Figure 3). Additionally,

For peer review only

utilization of most services reduced the most in December 2020 coinciding with start of health care workers strike, after which utilization of most services started to go back to expected levels.

--Table 2--

We fitted interrupted time series models for indicators that showed significant changes from the SPC charts. The rate ratios from the model are presented in Table 1. The month-to-month changes before COVID-19 were generally increasing across all the indicators. There was an immediate statistically significant reduction in all the indicators post-COVID-19, in the month immediately after first case, except for ANC 1 and new cases of diabetes and hypertension, which were unaffected. The statistically significant level changes post-COVID-19 were outpatient attendance for children under-fives which reduced by 50%, those for outpatients' over-fives by 35%, under-fives pneumonia outpatients by 43%, over-fives pneumonia outpatients by 38%, antenatal care 4th visit by 14%, total cases of diabetes by 5%, new cases of hypertension by 11% and HIV tests by 3%. There was a slight but statistically significant month-to-month increase in services post-COVID-19 (April 2020 to March 2021) of 5% for under-fives outpatients attendance, 2% for over-fives outpatients, 4% for under-fives pneumonia outpatients, 3% for over-fives pneumonia patients and no significant month-to-month changes for antenatal care visits, diabetes and hypertension cases. The trends from the fitted interrupted time series model are visually represented in Figure 4.

------------------------------------Figure 4--

Sensitivity analyses

Change point analysis showed the health workers' strike, which started in December 2020 had a significant impact on antenatal care 4th visits, and no effect on the other indicators (Additional File 5 SI Figure 1). Further, excluding the strike period (December 2020 to February 2021) from the segmented regression models of all indicators evaluated resulted in estimates that are not different from primary model estimates (Additional File 5 SI Table 1). Estimates from the Generalised estimating equations (GEE) models were not different from the primary model indicating robustness of reported estimates (Additional File 5 SI Table 2).

For peer review only

Discussion

Using DHIS2 health facility level monthly reported outpatient data, we provide evidence of COVID-19 impact on utilisation of basic health services in Kenya. The announcement of the first case of COVID-19 in Kenya in March 2020 and the intervention measures that followed coincided with sharp declines in outpatient and antenatal care fourth visits nationally. By the end of this study, health services are still in the process of returning to pre-COVID-19 levels. However, immunisation services remained unaffected.

Previous studies have found variable impacts on immunisation services,(17, 51, 52). In two studies that evaluated performance of routine immunization on selected indicators in Kenya, which used a relatively shorter period and didn't account for missing data, COVID-19 had no substantial impact on vaccination coverage, antenatal care first visits and a significant increase in measles immunization in March 2020 was reported,(17, 51). The significant increase in measles vaccines in March 2020 was due to increased immunization to make up for stock-out of measles vaccines between November 2019 and January 2020,(17). The sustained immunisation levels in the other antigens suggests there were no significant disruption to vaccine supply chain resulting from the pandemic, and confirmed by the National Vaccines and Immunisation Programme (NVIP),(17). Additionally, where health facilities designated as vaccination centres were assigned as COVID-19 isolation centres, the vaccines programme moved immunisation services to neighbouring health facilities,(17). These strategies illuminate why immunisation services remained unaffected during the pandemic, contrary to earlier predictions of reductions in immunization,(8, 9). Although not statistically significant, the slight reductions in the number of vaccines administered in December 2020 were likely attributed to the nationwide health worker strike, which led to staff shortages consequently affecting administration of the vaccines. These results strengthen previous findings with no observable differences in mean monthly number of immunisation and total antenatal care visits over a much shorter study period March-June 2020 relative to the same period in 2019 in Kenya,(52). Additionally, in a recent survey across 18 African countries, which evaluated disruption to essential health services in Africa during COVID-19, found that vaccination was the least disrupted service across all countries (30). In summary, immunisation services

For peer review only

were unaffected likely because of a number of reasons; the concerted effort by the NVIP to sustain supply of vaccines and unavailability of alternative sources for vaccination outside of the health system.

There were significant drops in nearly all outpatient services evaluated in this study. Total outpatient and pneumonia specific outpatient attendance were most affected, with utilization of the services dropping by half for under-fives. Moreover, COVID-19 had an impact on ANC 4, total attendance for hypertension and diabetes and HIV testing. Similar findings have been reported in other low- and middle-income countries,(16, 17, 19, 53-56). Studies evaluating the impact of lockdown measures to combat COVID-19 in South Africa observed a substantial drop in primary healthcare services utilisation,(16, 55). Significant drops in essential health services were also experienced following institution of public health measures to combat COVID-19 in Kinshasa, Democratic Republic of Congo,(19). Disruptions in general attendance have also been reported in various studies globally,(53, 57-60).

Various factors could explain the downward trends in specific outpatient services. In a survey conducted in Kenya to assess health services utilization during COVID-19, common causes reported by respondents include fear of risk of catching coronavirus at health facilities (26%), reduced incomes affecting ability to meet transport costs and other healthcare related costs (17%), shortage of healthcare workers in health facilities (14%), difficulties in accessing health facilities due to lockdowns and curfew (14%) and closing of some health facilities (14%),(61). The substantial declines for under-fives attendance are likely associated with reduced mixing due to closure of schools, improved hygiene practices and parents choosing to manage non-severe illnesses at home. Although attendance for ANC 4 was affected, it is unclear why the first visits were not affected. Notwithstanding, this might suggest that pregnant women attach greater importance to the primary ANC visit as has been reported,(62, 63) and hence despite the prevailing conditions managed to prioritize at least one visit to a health facility. Additionally, data has suggested deliveries in health facilities were also not affected during the pandemic (Wambua et al 2021, The indirect impact of COVID-19 pandemic on inpatient admissions in 204 Kenyan hospitals: An interrupted time series analysis), and this likely suggests the population of pregnant women remained relatively comfortable to use health services despite the pandemic.

For peer review only

A survey in Ethiopia among diabetic and hypertensive patients reported unavailability, unaffordable or increased price of medications and interruptions in follow-up visits were common barriers to accessing chronic care units in public facilities during the pandemic,(64). Reduction in attendance for chronic conditions such as hypertensive cases is a significant finding as missing care for these chronic illnesses could lead to further complications and susceptibility to severe COVID-19,(65) and increased morbidity and mortality. The gradual decline in HIV testing pre- COVID-19 might suggest reduced coverage due to policies geared towards targeted testing as opposed to blanket testing,(66). Additionally increased uptake and accessibility to testing in pharmacies implemented in 2017 might be associated with reduced testing in health facilities,(67). Pre-existing challenges in access to health services such as poor road network, disruptions in supplies to health facilities, and limited or no capacity for domestic production of medical supplies could have compounded the dramatic downward trends in utilisation of outpatient services. Additionally, improved hand hygiene and use of face masks during the pandemic could have led to reduced risk of other infectious diseases and consequently fewer visits to health facilities,(68, 69).

Strengths and implications of the study

Although most of the public attention is on control measures of COVID-19, possible health consequences from the indirect effects of the measures should not be overlooked. We provide a comprehensive understanding of the present situation on utilisation of immunisation and outpatient services in Kenya. Although the findings provide short-term estimates on the effect of COVID-19 at national level, studies could assess the long-term and differential effects at sub-national level. We addressed possible confounders in assessing changes overtime. For instance, in line with a recent guide on using routine data to monitor the effects of COVID-19 by the WHO, we adjusted for missing data which would have affected the validity of the comparisons over time,(70). Additionally, incompleteness may lead to biased estimates and strategies to improve data quality in DHIS2 such as investment in

For peer review only

better infrastructure, supervisory support, formal data quality assurance and human resources could improve reporting in Kenyan health facilities,(71, 72). We also use sensitivity analysis to account for any uncertainty in the estimates due to other factors affecting utilisation of services such as healthcare workers strikes and health-facility specific variations, which reduced bias and improved precision of the estimates.

Limitations and Recommendations

In this study, controls were not used to differentiate the impact of COVID-19 from other possible causes of the changes as most indicators were indirectly affected by the pandemic. However, since the drops in utilisation of services coincided with the introduction of COVID-19 intervention measures, the changes are attributed to COVID-19. We suggest sensitivity studies in future to assess any departures from the missing at random assumption when using multiple imputation for DHIS 2 data.

Conclusion

In summary, COVID-19 pandemic has had varied indirect effects on utilisation of outpatient health services. Although utilisation of immunisation services remained unchanged, there was a significant negative impact on outpatient clinic and ANC visits nationally. Total outpatient attendances for children under-fives reduced by 50%, under-fives pneumonia presentations reduced by 50%, general over-five visits reduced by 35%, over-fives pneumonia reduced by 38%, ANC 4 visits reduced by 14%, total hypertension cases reduced by 11%, total diabetes cases reduced by 5% and HIV testing by 3%. There is need for proactive and targeted interventions to avert and reverse these effects in future pandemics. These include strict implementation of safe practices and infrastructural changes in health facilities to reassure the public that it's safe to go to health facilities. Other innovative measures such as safe modes of transport, mobile clinics and supplementary immunisation activities (SIAs) could be incorporated in the pandemic response to avert any negative effects on utilisation of essential health services.

For peer review only

Declarations

This manuscript was submitted for publication with the permission of the Director KEMRI.

Funding

This research was funded in whole or in part by the Wellcome Trust Intermediate Fellow [Grant No. 201866]. For the purpose of Open Access, the author has applied a CC-BY public copyright licence to any author accepted manuscript version arising from this submission.

SW and **EAO** are supported through a Wellcome Trust Intermediate Fellow [Grant No. 201866]. **LM**, **JK, AN**, **GM**, **TT** and **CP** are supported through Funds from the Wellcome Trust [Grant No. 207522] awarded to Prof. Mike English (**ME**) as a senior Fellowship together with additional funds from a Wellcome Trust core grant awarded to the KEMRI-Wellcome Trust Research Programme [Grant No. 092654]. SW, LM, GM, TT, ME and EAO acknowledge the support of the Wellcome Trust to the Kenya Major Overseas Programme [Grant No. 203077].

Contributors

SW: Conceptualisation; Data curation; Formal analysis; Investigation; Methodology; Software; Validation; Visualisation; Writing - original draft. **LM**: Data curation; Formal analysis; Investigation; Methodology; Software; Validation; Visualisation; Writing - review & editing. **GM**: Data curation; Investigation; Software; Validation; Visualisation; Writing - review & editing. **AN**: Data curation; Formal analysis; Investigation; Software; Validation; Visualisation; Writing - review & editing. **JK**: Data curation; Formal analysis; Investigation; Software; Validation; Visualisation; Writing - review & editing. **TT**: Data curation; Investigation; Validation; Writing - review & editing **CP**: Data curation; Investigation; Validation; Writing - review & editing **ME**: Data curation; Investigation; Validation; Writing - review & editing. **EAO**: Data curation; Investigation; Validation; Funding acquisition; Writing - review & editing.

Data availability statement

Aggregated DHIS2 data is available online with access provided by Ministry of Health https://hiskenya.org/dhis-web-commons/security/login.action.

For peer review only

Ethics approval and consent to participate

The study does not contain any individual person's data.

Consent for publication

Not applicable.

Competing interests

The authors declare that they have no competing interests

Patient and public involvement

We have used secondary aggregated routine health information data available online and did not directly engage patients.

For peer review only

Figure and table legend

Table 1: Description of indicators evaluated in this study and the Kenyan Ministry of Health source forms used to capture the data.

Table 2: Segmented regression results showing rate ratios (R.R.) for COVID-19 intervention, time (ore-existing trend) and post-COVID-19 trend alongside 95% confidence intervals (CI) and p-values. The ARMA parameters (1,0) for ANC 1 and (2,0) for HIV tests performed where autocorrelation was detected are also provided.

Figure 1: Daily seven moving average trend of COVID-19 cases in Kenya showing various public health interventions initiated by the government to control the spread of the pandemic

Figure 2: Temporal trends in monthly immunisation and outpatient attendance nationally and by year

Figure 3: Statistical Process Control (SPC) chart of immunisation, antenatal care and outpatient services. Horizontal dashed lines represent the 3-standard deviation mark.

Figure 4: Fitted lines of interrupted timeseries models for outpatient and antenatal care attendance. Vertical lines represent the month (March 2020) COVID-19 was announced in Kenya and as a pandemic by the World Health Organization.

Supplementary Files

Additional File 1: Visual distribution patterns of missing data across all the health facilities analysed from DHIS 2

Additional File 2: Multiple Imputation model specification

Additional File 3: Number of health facilities analysed for each indicator including health facilities excluded for not reporting any month and those with less than 30% of months reported.

Additional File 4: Model selection information

For peer review only

Additional File 5: Sensitivity analyses model estimates

References

1. John Hopkins University. COVID-19 Dashboard [Available from: https://coronavirus.jhu.edu/.
2. Sam SO, Pokhariyal GP, Rogo K, Ndhine EO. Otoi-NARIMA model for forecast seasonality of COVID-19 waves: Case of Kenya. 2021.
3. Uyoga S, Adetifa IM, Karanja HK, Nyagwange J, Tuju J, Wanjiku P, et al. Seroprevalence of anti–SARS-CoV-2 IgG antibodies in Kenyan blood donors. Science. 2021;371(6524):79-82.
4. WHO. Kenya receives COVID-19 vaccines and launches landmark national campaign 2021 [01/07/2021]. Available from: https://www.afro.who.int/news/kenya-receives-covid-19-vaccines-and-launches-landmark-national-campaign.
5. Bai HM, Zaid A, Catrin S, Ahmed K, Ahmed A. The socio-economic implications of the coronavirus pandemic (COVID-19): A review. Int J Surg. 2020;8(4):8-17.
6. Quaife M, Van Zandvoort K, Gimma A, Shah K, McCreesh N, Prem K, et al. The impact of COVID-19 control measures on social contacts and transmission in Kenyan informal settlements. BMC medicine. 2020;18(1):1-11.
7. Parpia AS, Ndeffo-Mbah ML, Wenzel NS, Galvani AP. Effects of response to 2014–2015 Ebola outbreak on deaths from malaria, HIV/AIDS, and tuberculosis, West Africa. Emerging infectious diseases. 2016;22(3):433.
8. Riley T, Sully E, Ahmed Z, Biddlecom A. Estimates of the potential impact of the COVID-19 pandemic on sexual and reproductive health in low-and middle-income countries. International Perspectives on Sexual and Reproductive Health. 2020;46:73-6.
9. Roberton T, Carter ED, Chou VB, Stegmuller AR, Jackson BD, Tam Y, et al. Early estimates of the indirect effects of the COVID-19 pandemic on maternal and child mortality in low-income and middle-income countries: a modelling study. The Lancet Global Health. 2020;8(7):e901-e8.
10. Barden-O'Fallon J, Barry MA, Brodish P, Hazerjian J. Rapid assessment of Ebola-related implications for reproductive, maternal, newborn and child health service delivery and utilization in Guinea. PLoS currents. 2015;7.
11. Bolkan HA, van Duinen A, Samai M, Gassama I, Waalewijn B, Wibe A, et al. Admissions and surgery as indicators of hospital functions in Sierra Leone during the west-African Ebola outbreak. BMC health services research. 2018;18(1):846.
12. Brolin Ribacke KJ, van Duinen AJ, Nordenstedt H, Höijer J, Molnes R, Froseth TW, et al. The Impact of the West Africa Ebola Outbreak on Obstetric Health Care in Sierra Leone. PLoS One. 2016;11(2).

For peer review only

13. Elston J, Moosa A, Moses F, Walker G, Dotta N, Waldman RJ, et al. Impact of the Ebola outbreak on health systems and population health in Sierra Leone. Journal of Public Health. 2016;38(4):673-8.
14. Takahashi S, Metcalf CJE, Ferrari MJ, Moss WJ, Truelove SA, Tatem AJ, et al. Reduced vaccination and the risk of measles and other childhood infections post-Ebola. Science. 2015;347(6227):1240-2.
15. Chang H-J, Huang N, Lee C-H, Hsu Y-J, Hsieh C-J, Chou Y-J. The impact of the SARS epidemic on the utilization of medical services: SARS and the fear of SARS. American journal of public health. 2004;94(4):562-4.
16. Siedner MJ, Kraemer JD, Meyer MJ, Harling G, Mngomezulu T, Gabela P, et al. Access to primary healthcare during lockdown measures for COVID-19 in rural South Africa: an interrupted time series analysis. BMJ open. 2020;10(10):e043763.
17. Barasa E, Kazungu J, Orangi S, Kabia E, Ogero M, Kasera K. Assessing the Indirect Health Effects of the COVID-19 Pandemic in Kenya. Center for Global Development Forthcoming at https://www cgdev org/publication/assessing-indirect-health-effects-covid-19-pandemic-kenya. 2021.
18. McQuaid C, Vassall A, Cohen T, Fiekert K, White R. The impact of COVID-19 on TB: a review of the data. The International Journal of Tuberculosis and Lung Disease. 2021;25(6):436-46.
19. Hategeka C, Carter SE, Chenge FM, Katanga EN, Lurton G, Mayaka SM-N, et al. Impact of the COVID-19 pandemic and response on the utilisation of health services during the first wave in Kinshasa, the Democratic Republic of the Congo. medRxiv. 2021.
20. Kenya Go. National Emergency Response Committee on Coronavirus 2020 [cited 2020. Available from: https://www.health.go.ke/wp-content/uploads/2020/06/Executive-Order-No-2-of-2020_National-Emergency-Response-Committee-on-Coronavirus-28.2.20.pdf.
21. Health Mo. COVID-19 press releases by Health Ministry Health Ministry website2020 [cited 2020. Available from: https://www.health.go.ke/press-releases/.
22. Barasa E, Kazungu J, Orangi S, Kabia E, Ogero M, Kasera K. Assessing the Indirect Health Effects of the COVID-19 Pandemic in Kenya. CGD Work Pap. 2021;570.
23. Manya A, Braa J, Øverland LH, Titlestad OH, Mumo J, Nzioka C, editors. National roll out of District Health Information Software (DHIS 2) in Kenya, 2011–Central server and Cloud based infrastructure. IST-Africa 2012 Conference Proceedings; 2012: IIMC International Information Management Corporation.
24. Dehnavieh R, Haghdoost A, Khosravi A, Hoseinabadi F, Rahimi H, Poursheikhali A, et al. The District Health Information System (DHIS2): A literature review and meta-synthesis of its strengths and operational challenges based on the experiences of 11 countries. Health Information Management Journal. 2019;48(2):62-75.
25. Irimu G, Ogero M, Mbevi G, Kariuki C, Gathara D, Akech S, et al. Tackling health professionals' strikes: an essential part of health system strengthening in Kenya. BMJ global health. 2018;3(6).
26. Kwak SK, Kim JH. Statistical data preparation: management of missing values and outliers. Korean journal of anesthesiology. 2017;70(4):407.
27. WHO. Data quality review: a toolkit for facility data quality assessment. Module 2. Desk review of data quality 2017 [Available from: https://apps.who.int/iris/bitstream/handle/10665/259225/9789241512732-eng.pdf?sequence=1&isAllowed=y.
28. Kiberu VM, Matovu JK, Makumbi F, Kyozira C, Mukooyo E, Wanyenze RK. Strengthening district-based health reporting through the district health management information software system: the Ugandan experience. BMC medical informatics and decision making. 2014;14(1):1-9.
29. Begum T, Khan SM, Adamou B, Ferdous J, Parvez MM, Islam MS, et al. Perceptions and experiences with district health information system software to collect and utilize health data in Bangladesh: a qualitative exploratory study. BMC health services research. 2020;20:1-13.

For peer review only

30. Tuti T, Bitok M, Malla L, Paton C, Muinga N, Gathara D, et al. Improving documentation of clinical care within a clinical information network: an essential initial step in efforts to understand and improve care in Kenyan hospitals. BMJ Global Health. 2016;1(1):e000028.
31. Rubin DB. Inference and missing data. Biometrika. 1976;63(3):581-92.
32. Rubin DB. Multiple imputation after 18+ years. Journal of the American statistical Association. 1996;91(434):473-89.
33. Quartagno M. Multiple Imputation for Individual Patient Data Meta-Analyses: London School of Hygiene & Tropical Medicine; 2016.
34. Feng S, Hategeka C, Grépin KA. Addressing missing values in routine health information system data: an evaluation of imputation methods using data from the Democratic Republic of the Congo during the COVID-19 pandemic. 2021.
35. Quartagno M, Carpenter J, Quartagno MM, BaBooN S. Package 'jomo'. 2020.
36. Quartagno M, Grund S, Carpenter J. Jomo: a flexible package for two-level joint modelling multiple imputation. R Journal. 2019;9(1).
37. Benneyan JC. The design, selection, and performance of statistical control charts for healthcare process improvement. International Journal of Six Sigma and Competitive Advantage. 2008;4(3):209-39.
38. Killick R, Eckley I. changepoint: An R package for changepoint analysis. Journal of statistical software. 2014;58(3):1-19.
39. Taylor WA. Change-point analysis: a powerful new tool for detecting changes. 2000.
40. Wagner AK, Soumerai SB, Zhang F, Ross-Degnan D. Segmented regression analysis of interrupted time series studies in medication use research. Journal of clinical pharmacy and therapeutics. 2002;27(4):299-309.
41. Bernal JL, Cummins S, Gasparrini A. Interrupted time series regression for the evaluation of public health interventions: a tutorial. International journal of epidemiology. 2017;46(1):348-55.
42. Gardner W, Mulvey EP, Shaw EC. Regression analyses of counts and rates: Poisson, overdispersed Poisson, and negative binomial models. Psychological bulletin. 1995;118(3):392.
43. Ver Hoef JM, Boveng PL. Quasi-Poisson vs. negative binomial regression: how should we model overdispersed count data? Ecology. 2007;88(11):2766-72.
44. Bozdogan H. Model selection and Akaike's information criterion (AIC): The general theory and its analytical extensions. Psychometrika. 1987;52(3):345-70.
45. Prais SJ, Winsten CB. Trend estimators and serial correlation. Cowles Commission discussion paper Chicago; 1954.
46. Nelson BK. Time series analysis using autoregressive integrated moving average (ARIMA) models. Academic emergency medicine. 1998;5(7):739-44.
47. Schaffer AL, Dobbins TA, Pearson S-A. Interrupted time series analysis using autoregressive integrated moving average (ARIMA) models: a guide for evaluating large-scale health interventions. BMC medical research methodology. 2021;21(1):1-12.
48. Masarotto G, Varin C. Gaussian copula regression in R. Journal of Statistical Software. 2017;77(1):1-26.
49. Bhaskaran K, Gasparrini A, Hajat S, Smeeth L, Armstrong B. Time series regression studies in environmental epidemiology. International journal of epidemiology. 2013;42(4):1187-95.
50. Rubin DB. Multiple imputation for nonresponse in surveys: John Wiley & Sons; 2004.
51. Masresha BG, Luce Jr R, Shibeshi ME, Ntsama B, N'Diaye A, Chakauya J, et al. The performance of routine immunization in selected African countries during the first six months of the COVID-19 pandemic. The Pan African Medical Journal. 2020;37(Suppl 1).
52. SHIKUKU DN, Nyaoke I, Gichuru S, Maina O, Eyinda M, Godia P, et al. Early indirect impact of COVID-19 pandemic on utilization and outcomes of reproductive, maternal, newborn, child and adolescent health services in Kenya. medRxiv. 2020.

For peer review only

53. Burt JF, Ouma J, Amone A, Aol L, Sekikubo M, Nakimuli A, et al. Indirect Effects of COVID-19 on Maternal, Neonatal, Child, Sexual and Reproductive Health Services in Kampala, Uganda. medRxiv. 2021.
54. Dorward J, Khubone T, Gate K, Ngobese H, Sookrajh Y, Mkhize S, et al. The impact of the COVID-19 lockdown on HIV care in 65 South African primary care clinics: an interrupted time series analysis. The Lancet HIV. 2021;8(3):e158-e65.
55. Adelekan T, Mihretu B, Mapanga W, Nqeketo S, Chauke L, Dwane Z, et al. Early effects of the COVID-19 pandemic on family planning utilisation and termination of pregnancy services in Gauteng, South Africa: March–April 2020. Wits Journal of Clinical Medicine. 2020;2(2):145-52.
56. Wanyana D, Wong R, Hakizimana D. Rapid assessment on the utilization of maternal and child health services during COVID-19 in Rwanda. Public Health Action. 2021;11(1):12-21.
57. Chanchlani N, Buchanan F, Gill PJ. Addressing the indirect effects of COVID-19 on the health of children and young people. CMAJ. 2020;192(32):E921-E7.
58. Franchini S, Spessot M, Landoni G, Piani C, Cappelletti C, Mariani F, et al. Stranger months: how SARS-CoV-2, fear of contagion, and lockdown measures impacted attendance and clinical activity during February and March 2020 at an urban Emergency Department in Milan. Disaster medicine and public health preparedness. 2020:1-10.
59. Migliori GB, Thong PM, Akkerman O, Alffenaar J-W, Álvarez-Navascués F, Assao-Neino MM, et al. Worldwide effects of coronavirus disease pandemic on tuberculosis services, January–April 2020. Emerging infectious diseases. 2020;26(11):2709.
60. Murewanhema G, Makurumidze R. Essential health services delivery in Zimbabwe during the COVID-19 pandemic: perspectives and recommendations. The Pan African Medical Journal. 2020;35(Suppl 2).
61. Partnership for Evidence-Based Response to COVID-19 (PERC). Using data to find a balance,Special report series: Disruption to essential health services in Africa during COVID-19 2020 [cited 2021 21/06/2021]. Available from: https://preventepidemics.org/wp-content/uploads/2020/11/PERC-Brief-Essential-Services_Report_1120.pdf.
62. Edie GEHE, Obinchemti TE, Tamufor EN, Njie MM, Njamen TN, Achidi EA. Perceptions of antenatal care services by pregnant women attending government health centres in the Buea Health District, Cameroon: a cross sectional study. Pan African Medical Journal. 2015;21(1).
63. Brown CA, Sohani SB, Khan K, Lilford R, Mukhwana W. Antenatal care and perinatal outcomes in Kwale district, Kenya. BMC pregnancy and childbirth. 2008;8(1):1-11.
64. Shimels T, Asrat Kassu R, Bogale G, Bekele M, Getnet M, Getachew A, et al. Magnitude and associated factors of poor medication adherence among diabetic and hypertensive patients visiting public health facilities in Ethiopia during the COVID-19 pandemic. PloS one. 2021;16(4):e0249222.
65. Apicella M, Campopiano MC, Mantuano M, Mazoni L, Coppelli A, Del Prato S. COVID-19 in people with diabetes: understanding the reasons for worse outcomes. The lancet Diabetes & endocrinology. 2020.
66. Agutu CA, Oduor TH, Kombo BK, Mugo PM, Chira SM, Ogada FW, et al. High patient acceptability but low coverage of provider-initiated HIV testing among adult outpatients with symptoms of acute infectious illness in coastal Kenya. PloS one. 2021;16(2):e0246444.
67. Mugo PM, Micheni M, Shangala J, Hussein MH, Graham SM, Rinke de Wit TF, et al. Uptake and acceptability of oral HIV self-testing among community pharmacy clients in Kenya: a feasibility study. PloS one. 2017;12(1):e0170868.
68. Aiello AE, Coulborn RM, Perez V, Larson EL. Effect of hand hygiene on infectious disease risk in the community setting: a meta-analysis. American journal of public health. 2008;98(8):1372-81.
69. Liang M, Gao L, Cheng C, Zhou Q, Uy JP, Heiner K, et al. Efficacy of face mask in preventing respiratory virus transmission: A systematic review and meta-analysis. Travel medicine and infectious disease. 2020;36:101751.

For peer review only

70. WHO. Analysing and using routine data to monitor the effects of COVID-19 on essential health services 2021 [01/06/2021]. Available from: https://www.who.int/bulletin/volumes/95/10/17-194399/en/.
71. Hagel C, Paton C, Mbevi G, English M. Data for tracking SDGs: challenges in capturing neonatal data from hospitals in Kenya. BMJ Global Health. 2020;5(3):e002108.
72. Kihuba E, Gathara D, Mwinga S, Mulaku M, Kosgei R, Mogoa W, et al. Assessing the ability of health information systems in hospitals to support evidence-informed decisions in Kenya. Global health action. 2014;7(1):24859.

For peer review only

Table 1: Description of indicators analysed in this study and Kenyan Ministry of Health (MOH) source forms used to capture the data.

Category	Description	Assigned names in this study	Source form
Immunization	BCG vaccine doses administered	BCG	MOH 710
	Oral polio vaccine doses administered	OPV dose 1, dose 2 & dose 3	MOH 710
	Rotavirus vaccine doses administered	Rotavirus dose 1 & dose 2	MOH 710
	Pneumococcal conjugate vaccine doses administered	Pneumococcal dose 1, dose 2 & dose 3	MOH 710
	DPT vaccine doses administered	DPT 1, 2 & 3	MOH 710
	Inactivated polio vaccine doses administered	IPV	MOH 710
	Measles vaccine doses administered	Measles dose 1 & dose 2	MOH 710
Outpatient visits	Antenatal care first visit	ANC 1	MOH 711
	Antenatal care fourth visits	ANC 4	MOH 711
	Outpatient department visits in under-fives	OPD < 5 years	MOH 705A
	Outpatient department visits in over-fives	OPD > 5 years	MOH 705B
	Outpatient department visits with pneumonia in under-fives	OPD Pneumonia < 5 years	MOH 705 A
	Outpatient department visits with pneumonia in over-fives	OPD Pneumonia > 5 years	MOH 705B
	Number of new cases of diabetes	Diabetes new cases	MOH 705 A & B
	Number of new plus revisits of diabetes cases	Diabetes total cases	MOH 705 A & B
	Number of new hypertension cases	Hypertension new cases	MOH 705 A & B
	Number of new plus revisits of hypertension cases	Hypertension total cases	MOH 705 A & B
	Number of HIV tests performed	HIV tests performed	MOH 731

For peer review only

Table 2: Segmented regression results showing rate ratios (RR) for COVID-19 intervention, time (pre-existing trend) and trend (post-COVID-19 trend). The 95% confidence intervals and p-values are also show.

Covariate	OPD < 5 years			OPD > 5 years			OPD Pneumonia < 5 years			OPD Pneumonia > 5 years		
	RR	95%CI	P-value	RR	95%CI	P-value	RR	95%CI	P-value	RR	95%CI	P-value
COVID-19	0.50	(0.44-0.57)	<0.01	0.65	(0.57-0.75)	<0.01	0.43	(0.38-0.47)	<0.01	0.62	(0.55-0.70)	<0.01
Time	1.00	(0.99-1.01)	0.15	1.00	(1.00-1.01)	0.02	1.01	(1.00-1.01)	<0.01	1.00	(0.99-1.01)	0.05
Trend	1.05	(1.03-1.06)	<0.01	1.02	(1.00-1.04)	0.03	1.07	(1.05-1.08)	<0.01	1.03	(1.02-1.05)	<0.01
	ANC 1			ANC 4			Diabetes new cases			Diabetes total cases		
	RR*	95%CI*	P-value*	RR	95%CI	P-value	RR	95%CI	P-value	RR	95%CI	P-value
COVID-19	0.96	(0.83-1.10)	0.55	0.86	(0.80-0.93)	<0.01	1.17	(0.89-1.52)	0.25	0.95	(0.93-0.97)	<0.01
Time	1.00	(0.99-1.00)	0.61	1.00	(0.99-1.00)	0.13	0.99	(0.98-1.00)	0.13	1.00	(1.00-1.01)	<0.01
Trend	1.01	(0.99-1.03)	0.12	1.00	(0.99-1.01)	0.90	0.99	(0.97-1.01)	0.57	1.00	(1.00-1.00)	0.05
	Hypertension new cases			Hypertension total cases			HIV Tests Performed					
	RR	95%CI	P-value	RR	95%CI	P-value	RR*	95%CI*	P-value*			
COVID-19	0.87	(0.75-1.00)	0.05	0.89	(0.82-0.96)	<0.01	0.97	(0.94-0.99)	0.01			
Time	1.00	(0.99-1.01)	0.81	1.01	(1.00-1.01)	<0.01	0.97	(0.97-0.97)	<0.01			
Trend	1.00	(0.99-1.01)	0.59	1.00	(0.99-1.01)	0.90	1.01	(1.00-1.01)	<0.01			

*Estimate after fitting ARMA model to indicator where autocorrelation was detected. The ARMA (p, q) parameters for ANC 1 are (1,0) and (2,0) for HIV tests performed.

For peer review only - http://bmjopen.bmj.com/site/about/guidelines.xhtml

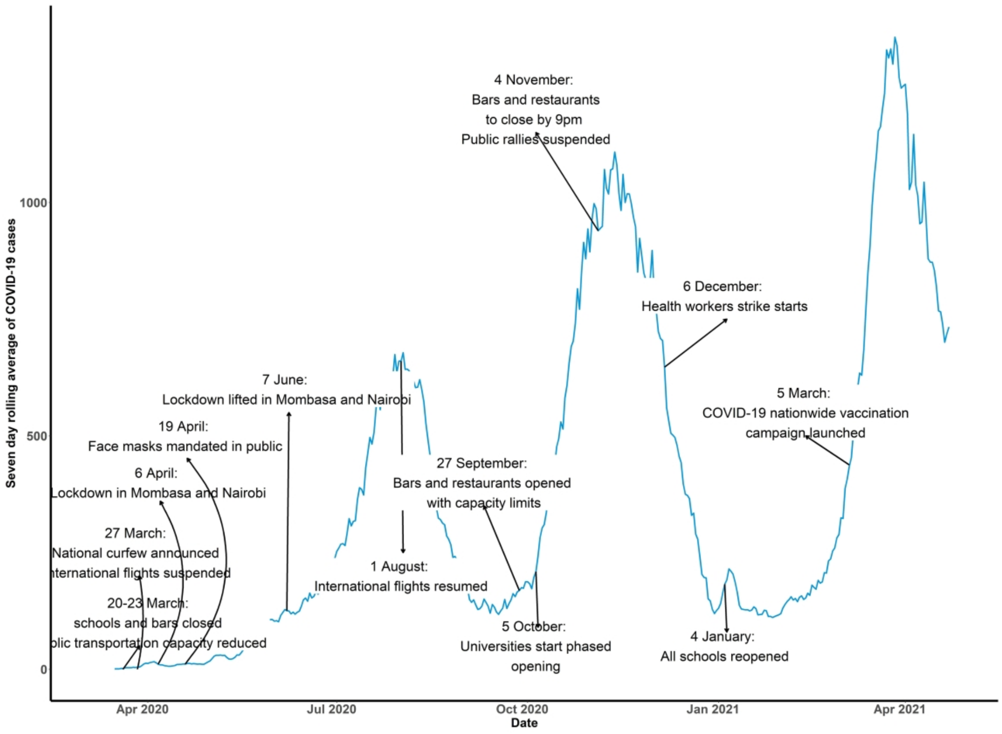

Daily seven moving average trends of COVID-19 cases in Kenya showing various interventions initiated by the government.

190x142mm (500 x 500 DPI)

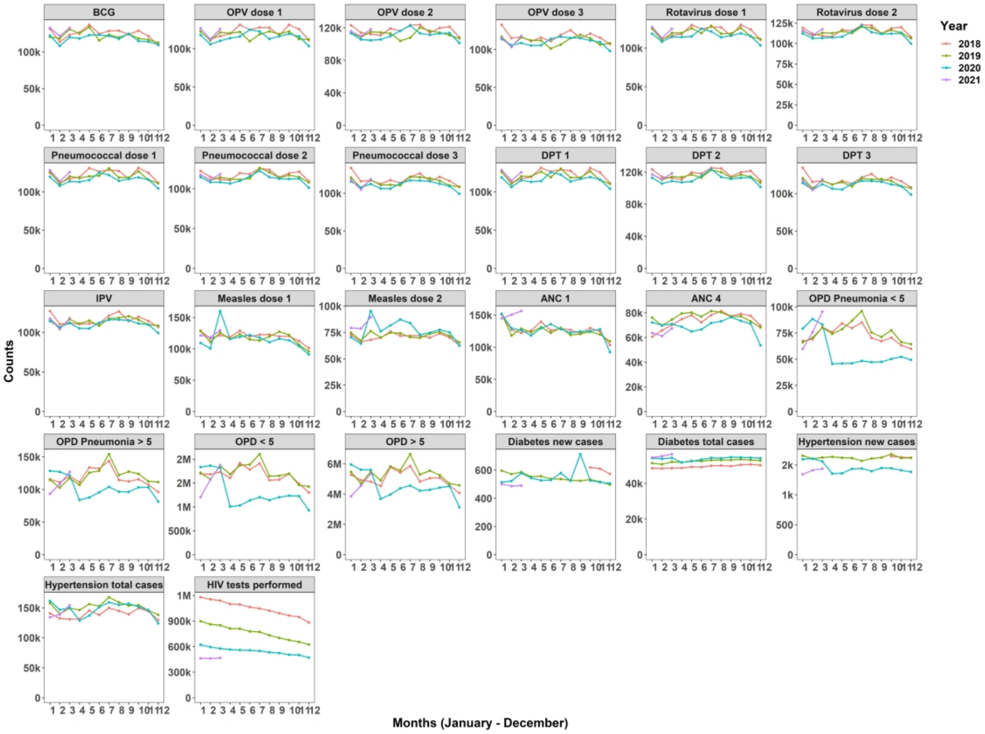

Temporal trends in monthly immunisation and outpatient attendance nationally and by year

190x142mm (500 x 500 DPI)

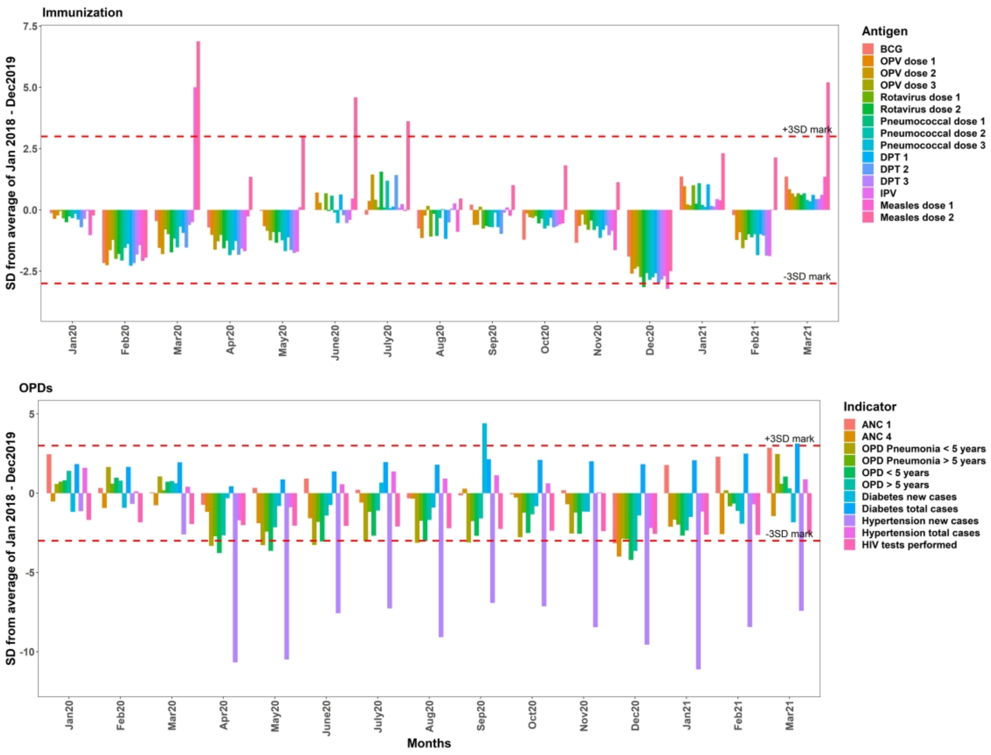

Statistical Process Control chart of immunisation, antenatal care and outpatient services. Horizontal dashed lines represent the 3-standard deviation mark.

190x142mm (500 x 500 DPI)

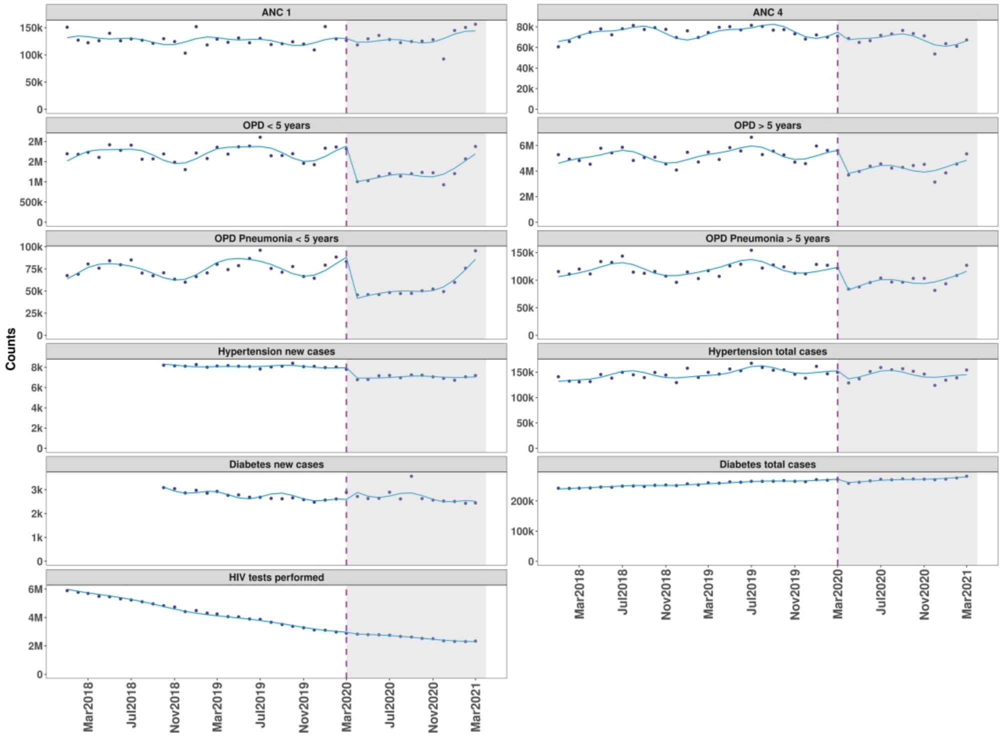

Fitted lines of segmented regression models for outpatient and antenatal care attendance. Vertical lines represent the month (March 2020) COVID-19 was announced in Kenya and as a pandemic by the WHO.

190x142mm (500 x 500 DPI)

SI Figure 1: Missing data patterns plot for indicators showing number of reported months by health facilities.

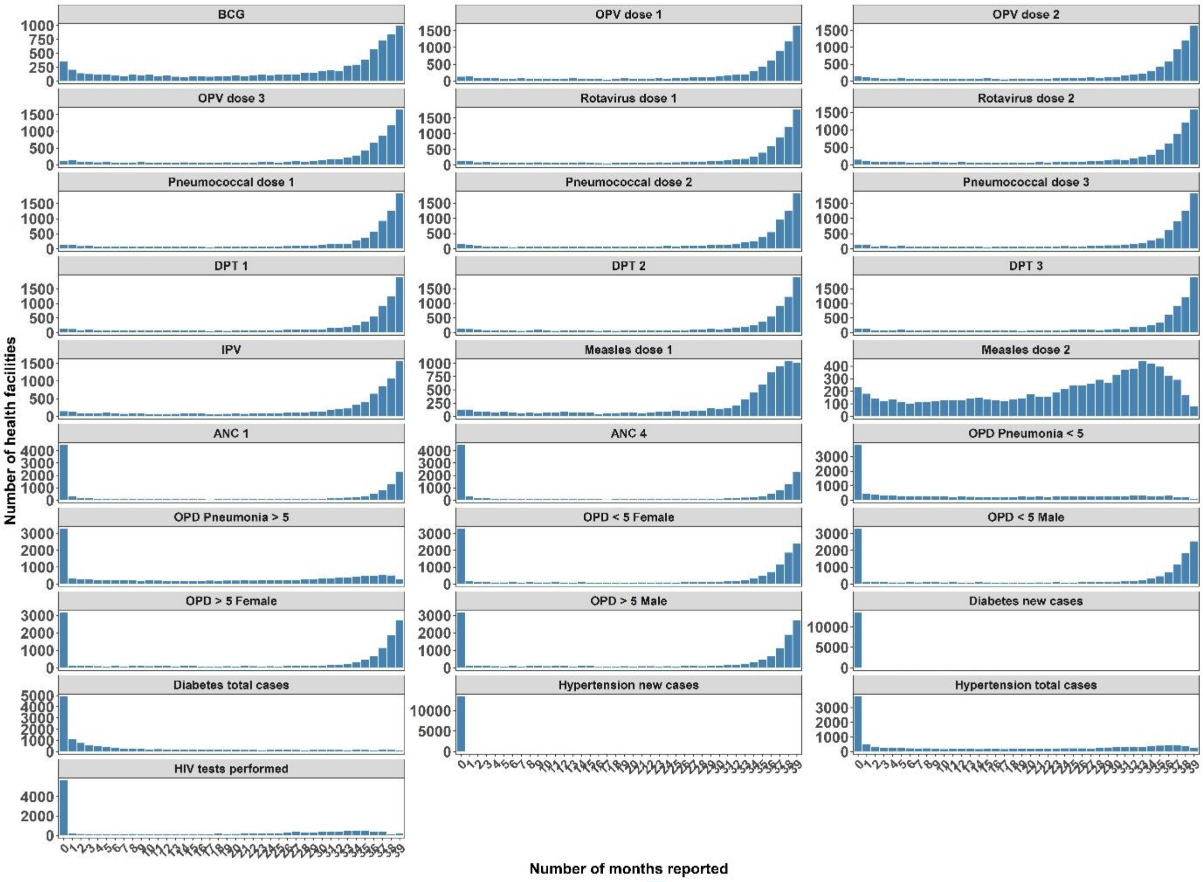

The x – axis shows the number of months reported by health facilities (0 to 39). 0 to the left means the health facilities did not report any month or may not be offering the service, while 39 means the health facilities reported all months.

Additional Information 4

Handling missing data

Missing data occurrence

Missing data occurred for the indicators in a given month for a given health facility.

Health facility ownership (public or private), level of health facility, time (month and year) and COVID-19 binary indicator (0 – months before pandemic and 1 – months post pandemic) were used as covariates (independent variables) with the health facility as a clustering variable. These hospital characteristics were fully observed across all hospitals.

Multiple Imputation under joint modelling framework

We implemented MI under the joint modelling imputation framework. In instances with complete data, standard statistical approaches for multi-level data apply models accounting for this dependency (1). Similarly, for missing values, imputation techniques need to account for dependency between observations, otherwise the predictive variance of the missing data is not accurately reflected. Certainly, if an incomplete variable is imputed ignoring the multilevel structure, the resultant imputations can be unreliable (1), and consequently bias in estimates obtained from imputed data. Therefore, to account for the multilevel structure, imputation techniques based on regression models that include a random intercept for clusters are generally used and have been implemented in the R-package *jomo* (2-4).

General model specification under joint modelling framework

Since missing data was observed in each indicator (outcome variable) in our dataset, with hospital characteristics fully observed (covariates), this presents a univariate missing data pattern (1). The general imputation model under this scenario is outlined below (1);

Let the matrix $Y_{n\times p} = (y_1, \dots, y_p)$ be the matrix of incomplete data for n items in rows and p variables in columns. Let i be the index for the individuals (($1 \le i \le n$ and j for columns ($1 \le j \le p$). Y is stratified to K clusters of size n_k where k denotes the index for a cluster ($1 \le k \le K$). So, y_{jk} denotes the n_k- vector corresponding to vector y_j restricted to individuals within

For peer review only

cluster k. Then, let (y_j^{obs}, y_j^{miss}) be the missing and observed parts of y_j and let $Y^{obs} = (y_1^{obs}, \dots, y_p^{obs})$ and $Y^{miss} = (y_1^{miss}, \dots, y_p^{miss})$. The imputation draws missing values from the predictive distribution $P(Y^{miss}|Y^{obs})$, where, an imputation model with parameter θ is specified and realizations of the predictive distribution of the missing values can be obtained by; drawing θ from $P(\theta|Y^{obs})$ its posterior distribution and drawing missing values according to $P(Y^{miss}|Y^{obs}, \theta)$ their predictive distribution given θ (1). In our case of a single incomplete variable (y_p), the posterior distribution can be specified by letting $\theta = (\beta, \Psi, \sum_k)$ be the parameters of a linear mixed effects model (1):

$$y_{pk} = Z_k\beta + W_k b_k + \varepsilon_k, \quad (1)$$

$$b_k \sim N(0, \Psi), \quad (2)$$

$$\varepsilon_k \sim N(0, \textstyle\sum_k) \quad (3)$$

Where y_{pk} denotes the incomplete variable restricted to the cluster k, $Z_k(n_k \times q)$ and $W_k(n_k \times q')$ are the known covariate arrays corresponding to two subsets of $(y_{1k}, \dots, y(p-1)k)$, β is the q –vector of regression coefficients of fixed effects, b_k is the q'-vector of random effects for cluster k, $\Psi(q' \times q')$ is the between cluster variance matrix, and $\sum_k = \delta_k^2 I_{nk}(n_k \times n_k)$ is the variance matrix within cluster k.

Implementation of the model to DHIS2

Since the number of attendances is count, linear transformation was important before imputation, following an appropriate variance-stabilizing transformation to make the normal distribution assumption more plausible. The variance-stabilizing transformation for the Poisson distribution of count data is the square root, and it provides a better transformation relative to the log transformation for count data (5-9). The back transformed values under the square root method align with the original count scale. A linear mixed effects model was then selected for the implementation of MI. Below is a representation of the specified model in

For peer review only

matrix form:

$$Y_i = X_i\beta + Z_j b_j + \varepsilon_i \quad (4)$$

Where: Y_i is response vector of the indicators X_i the model matrix for the fixed effects (health facility covariates; Health facility ownership (public or private), level of health facility, COVID-19 binary indicator (0 – months before pandemic and 1 – months post pandemic) and time (time data was reported as months and year combined)) and Z_i the model matrix for the random intercept for observations in the jth health facility. The vector of health facility covariates coefficients is represented by β while b_j represents the vector of random-effect coefficients in health facility j . The errors terms denoted by ϵ_i are assumed to follow a multivariate normal distribution with mean vector 0 and variance covariance matrix ∑. The MI mixed effects model was implemented in R version 3.6.3 using the *jomo* package for multilevel imputation (10).

References

1. Audigier V, White IR, Jolani S, Debray TP, Quartagno M, Carpenter J, et al. Multiple imputation for multilevel data with continuous and binary variables. Statistical Science. 2018;33(2):160-83.
2. Quartagno M, Grund S, Carpenter J. Jomo: a flexible package for two-level joint modelling multiple imputation. R Journal. 2019;9(1).
3. Quartagno M, Carpenter J, Quartagno MM, BaBooN S. Package 'jomo'. 2020.
4. Quartagno M, Carpenter J. Multiple imputation for IPD meta-analysis: allowing for heterogeneity and studies with missing covariates. Stat Med. 2016;35(17):2938-54.
5. Quartagno M. Multiple Imputation for Individual Patient Data Meta-Analyses: London School of Hygiene & Tropical Medicine; 2016.
6. Yu G. Variance stabilizing transformations of Poisson, binomial and negative binomial distributions. Statistics & Probability Letters. 2009;79(14):1621-9.
7. Crawley MJ. The R book: John Wiley & Sons; 2012.
8. Maindonald J, Braun J. Data analysis and graphics using R: an example-based approach: Cambridge University Press; 2006.
9. Stroup WW. Rethinking the analysis of non-normal data in plant and soil science. Agron J. 2015;107(2):811-27.
10. Quartagno M, Grund S, Carpenter J. Jomo: a flexible package for two-level joint modelling multiple imputation. R Journal. 2019.

For peer review only

SI Table 1: Number and percentage of health facilities analysed for each indicator. It shows number of facilities that did not report any month and those that were imputed (health facilities with more than 30% of months reported)

Indicator	All hospitals expected to report in DHIS2	Number of health facilities imputed	Number of health facilities with no reported data	Percent of health facilities analysed out of those reporting at least a month
BCG	8063	6509	352	84
DPT1	8063	7142	130	90
DPT2	8063	7141	140	90
DPT3	8063	7136	124	90
IPV	8063	7089	144	90
Measles1	8063	7166	125	90
Measles2	8063	6578	230	84
OPV1	8063	7134	128	90
OPV2	8063	7144	140	90
OPV3	8063	7124	123	90
Pneum1	8063	7139	132	90
Pneum2	8063	7143	141	90
Pneum3	8063	7145	129	90
Rota1	8063	7126	130	90
Rota2	8063	7114	146	90
ANC 1	13595	7768	4450	85
ANC 4	13595	7768	4450	85
OPD > 5 Female	13595	9434	3156	90
OPD > 5 Male	13595	9431	3153	90
OPD < 5 Female	13595	9246	3274	90
OPD < 5 Male	13595	9250	3276	90
OPD Pneumonia > 5	13595	7933	3264	77
OPD Pneumonia < 5	13798	6976	3784	70
Diabetes new cases	13752	72	13472	26
Diabetes total cases	13752	4220	4914	48
Hypertension new cases	13752	121	13454	41
Hypertension total cases	13757	7381	3765	74
HIV tests performed	13752	6789	5674	84

For peer review only

Table 1: Intraclass correlation coefficient (ICC)

Indicator	ICC
OPD <5 years	0.72
OPD > 5 years	0.82
OPD Pneumonia > 5 years	0.52
OPD Pneumonia < 5 years	0.43
ANC 1	0.87
ANC 4	0.76
Diabetes new cases	0.51
Diabetes total cases	0.96
Hypertension new cases	0.93
Hypertension total cases	0.72
HIV tests	0.92

Table 2: Akaike Information Criterion (AIC)

Indicator	Negative Binomial model	Negative Binomial model accounting for seasonality
OPD <5 years	1061.42	1042.73
OPD > 5 years	1147.58	1138.40
OPD Pneumonia < 5 years	841.78	832.94
OPD Pneumonia > 5 years	849.94	846.07
ANC 1	858.49	857.58
ANC 4	791.49	763.62
Diabetes new cases	419.34	417.93
Diabetes total cases	731.45	730.82
Hypertension new cases	340.09	338.17
Hypertension total cases	830.11	819.39
HIV tests	1008.38	1005.78

For peer review only

Table 3: Segmented regression estimates for Negative binomial model before adjusting for seasonality

Covariate	OPD < 5 years			OPD > 5 years			OPD Pneumonia < 5 years			OPD Pneumonia > 5 years		
	RR	95%CI	P-value	RR	95%CI	P-value	RR	95%CI	P-value	RR	95%CI	P-value
COVID-19	0.56	(0.47-0.65)	<0.01	0.72	(0.62-0.84)	<0.01	0.49	(0.42-0.57)	<0.01	0.69	(0.59-0.82))	<0.01
Time	1.00	(1.00-1.01)	0.75	1.00	(1.00-1.01)	0.18	1.01	(1.00-1.01)	<0.01	1.00	(1.00-1.01)	0.46
Trend	1.04	(1.02-1.06)	<0.01	1.01	(0.99-1.03)	0.42	1.03	(1.01-1.05)	<0.01	1.00	(1.00-1.04)	0.05

	ANC 1			ANC 4			Diabetes new cases			Diabetes total cases		
	RR	95%CI	P-value	RR	95%CI	P-value	RR	95%CI	P-value	RR	95%CI	P-value
COVID-19	0.96	(0.83-1.10)	0.53	0.95	(0.84-1.08)	0.45	1.12	(0.89-1.41)	0.34	0.97	(0.95-0.98)	<0.01
Time	1.00	(0.99-1.00)	0.45	1.00	(1.00-1.01	0.52	0.99	(0.98-1.00)	0.14	1.01	(1.00-1.02)	<0.01
Trend	1.02	(1.00-1.03)	0.07	0.99	(0.97-1.01)	0.25	1.00	(0.99-1.02)	0.87	1.00	(0.99-1.00)	0.56

	Hypertension new cases			Hypertension total cases			HIV Tests Performed		
	RR	95%CI	P-value	RR	95%CI	P-value	RR	95%CI	P-value
COVID-19	0.92	(0.82-1.04)	0.21	0.92	(0.86-0.99)	0.03	1.01	(0.98-1.05)	0.41
Time	0.99	(0.99-1.01)	0.18	1.01	(1.00-1.01)	<0.01	0.97	(0.97-0.97)	<0.01
Trend	1.04	(0.99-1.10)	0.14	0.99	(0.98-1.00)	<0.01	1.00	(0.99-1.01)	0.69

For peer review only

SI Figure 1: Multiple change point analysis plots showing significant shifts in attendance

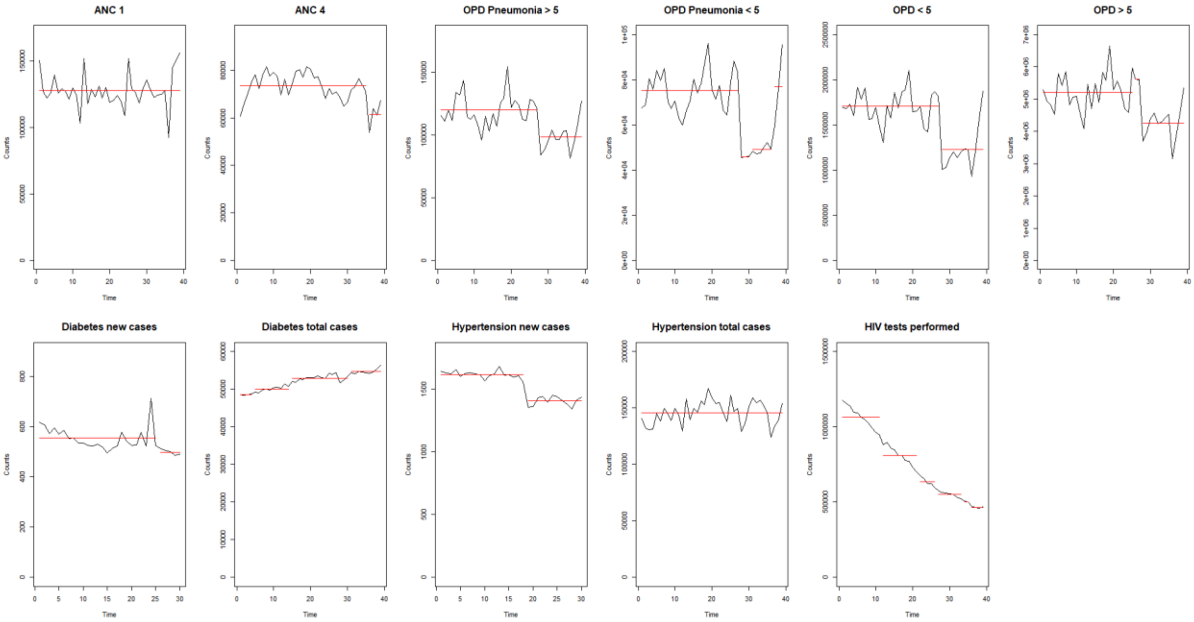

For peer review only

SI Table 1: Interrupted time series models comparing estimates before and after excluding strike period

		OPD < 5			OPD > 5			OPD Pneumonia < 5			OPD Pneumonia > 5		
**Ownership**		**RR**	**95%CI**	**P-value**	**RR**	**95%CI**	**P-value**	**RR**	**95%CI**	**P-value**	**RR**	**95%CI**	**P-value**
**Primary**	**COVID-19**	0.50	(0.44-0.57)	<0.01	0.65	(0.57-0.75)	<0.01	0.43	(0.38-0.47)	<0.01	0.62	(0.55-0.70)	<0.01
	**Time**	1.00	(0.99-1.01)	0.15	1.00	(1.00-1.01)	0.02	1.01	(1.00-1.01)	<0.01	1.00	(0.99-1.01)	0.05
	**Trend**	1.05	(1.03-1.06)	<0.01	1.02	(1.00-1.04)	0.03	1.07	(1.05-1.08)	<0.01	1.03	(1.02-1.05)	<0.01
**Excluding Strike**	**COVID-19**	0.45	(0.39-0.52)	<0.01	0.60	(0.53-0.68)	<0.01	0.39	(0.33-0.47)	<0.01	0.58	(0.52-0.66)	<0.01
	**Time**	1.00	(1.00-1.01)	0.13	1.01	(1.00-1.01)	<0.01	1.01	(1.00-1.01)	0.02	1.00	(1.00-1.01)	0.03
	**Trend**	1.09	(1.06-1.11)	<0.01	1.05	(1.03-1.07)	<0.01	1.10	(1.07-1.13)	<0.01	1.06	(1.04-1.08)	<0.01

		ANC 1			ANC 4			Diabetes new cases			Diabetes total cases		
		**RR**	**95%CI**	**P-value**	**RR**	**95%CI**	**P-value**	**RR**	**95%CI**	**P-value**	**RR**	**95%CI**	**P-value**
**Primary**	**COVID-19**	0.96	(0.83-1.10)	0.55	0.86	(0.80-0.93)	<0.01	1.17	(0.89-1.52)	0.25	0.95	(0.93-0.97)	<0.01
	**Time**	1.00	(0.99-1.00)	0.61	1.00	(0.99-1.00)	0.13	0.99	(0.98-1.00)	0.13	1.00	(1.00-1.01)	<0.01
	**Trend**	1.01	(0.99-1.03)	0.12	1.00	(0.99-1.01)	0.90	0.99	(0.97-1.01)	0.57	1.00	(1.00-1.00)	0.05
**Excluding Strike**	**COVID-19**	0.96	(0.84-1.09)	0.52	0.83	(0.77-0.89)	<0.01	1.12	(0.85-1.48)	0.43	0.94	(0.92-0.96)	<0.01
	**Time**	1.00	(1.00-1.00)	0.44	1.00	(1.00-1.00)	0.05	0.99	(0.98-1.00)	0.12	1.00	(1.00-1.01)	<0.01
	**Trend**	1.02	(1.00-1.04)	0.06	1.01	(1.00-1.03)	0.02	1.01	(0.98-1.04)	0.73	1.01	(1.00-1.01)	<0.01

		Hypertension new cases			Hypertension total cases			HIV Tests Performed		
		**RR**	**95%CI**	**P-value**	**RR**	**95%CI**	**P-value**	**RR**	**95%CI**	**P-value**
**Primary**	**COVID-19**	0.87	(0.75-1.00)	0.05	0.89	(0.82-0.96)	<0.01	0.97	(0.94-0.99)	0.01
	**Time**	1.00	(0.99-1.01)	0.81	1.01	(1.00-1.01)	<0.01	0.97	(0.97-0.97)	<0.01
	**Trend**	1.00	(0.99-1.01)	0.59	1.00	(0.99-1.01)	0.90	1.01	(1.01-1.01)	<0.01
**Excluding Strike**	**COVID-19**	0.86	(0.74-1.00)	0.06	0.85	(0.79-0.92)	<0.01	0.97	(0.94-1.01)	0.11
	**Time**	1.00	(0.99-1.01)	0.81	1.01	(1.00-1.01)	<0.01	0.97	(0.97-0.97)	<0.01
	**Trend**	1.01	(0.99-1.02)	0.48	1.01	(1.00-1.03)	0.02	1.01	(1.00-1.01)	<0.01

For peer review only

SI Table 2: Generalised estimating equations (GEE) results at health facility level showing rate ratios (RR) for COVID-19 intervention, time and trend alongside 95% confidence intervals for all indicators

		OPD < 5			OPD > 5			OPD Pneumonia < 5			OPD Pneumonia > 5		
**Ownership**		**RR**	**95%CI**	**P-value**	**RR**	**95%CI**	**P-value**	**RR**	**95%CI**	**P-value**	**RR**	**95%CI**	**P-value**
**Primary**	**COVID-19**	0.50	(0.44-0.57)	<0.01	0.65	(0.57-0.75)	<0.01	0.43	(0.38-0.47)	<0.01	0.62	(0.55-0.70)	<0.01
	**Time**	1.00	(0.99-1.01)	0.15	1.00	(1.00-1.01)	0.02	1.01	(1.00-1.01)	<0.01	1.00	(0.99-1.01)	0.05
	**Trend**	1.05	(1.03-1.06)	<0.01	1.02	(1.00-1.04)	0.03	1.07	(1.05-1.08)	<0.01	1.03	(1.02-1.05)	<0.01
**GEE**	**COVID-19**	0.50	(0.48-0.51)	<0.01	0.65	(0.64-0.66)	<0.01	0.42	(0.40-0.43)	<0.01	0.62	(0.60-0.64)	<0.01
	**Time**	1.00	(1.00-1.01)	<0.01	1.01	(1.00-1.01)	<0.01	1.01	(1.00-1.01)	<0.01	1.00	(1.00-1.01)	<0.01
	**Trend**	1.05	(1.04-1.05)	<0.01	1.02	(1.01-1.02)	<0.01	1.07	(1.06-1.07)	<0.01	1.03	(1.03-1.04)	<0.01
		**ANC 1**			**ANC 4**			**Diabetes new cases**			**Diabetes total cases**		
		**RR**	**95%CI**	**P-value**	**RR**	**95%CI**	**P-value**	**RR**	**95%CI**	**P-value**	**RR**	**95%CI**	**P-value**
**Primary**	**COVID-19**	0.96	(0.83-1.10)	0.55	0.86	(0.80-0.93)	<0.01	1.17	(0.89-1.52)	0.25	0.95	(0.93-0.97)	<0.01
	**Time**	1.00	(0.99-1.00)	0.61	1.00	(0.99-1.00)	0.13	0.99	(0.98-1.00)	0.13	1.00	(1.00-1.01)	<0.01
	**Trend**	1.01	(0.99-1.03)	0.12	1.00	(0.99-1.01)	0.90	0.99	(0.97-1.01)	0.57	1.00	(1.00-1.00)	0.05
**GEE**	**COVID-19**	0.96	(0.92-0.99)	0.01	0.87	(0.83-0.90)	<0.01	1.17	(0.89-1.53)	0.26	0.95	(0.93-0.98)	<0.01
	**Time**	1.00	(0.99-1.00)	0.05	1.00	(1.00-1.01)	0.01	0.99	(0.98-1.00)	0.13	1.00	(1.00-1.01)	<0.01
	**Trend**	1.01	(1.01-1.02)	<0.01	1.00	(0.99-1.01)	0.71	0.99	(0.98-1.01)	0.58	1.00	(1.00-1.00)	0.26
		**Hypertension new cases**			**Hypertension total cases**			**HIV Tests Performed**					
		**RR**	**95%CI**	**P-value**	**RR**	**95%CI**	**P-value**	**RR**	**95%CI**	**P-value**			
**Primary**	**COVID-19**	0.87	(0.75-1.00)	0.05	0.89	(0.82-0.96)	<0.01	0.97	(0.94-0.99)	0.01			
	**Time**	1.00	(0.99-1.01)	0.81	1.01	(1.00-1.01)	<0.01	0.97	(0.97-0.97)	<0.01			
	**Trend**	1.00	(0.99-1.01)	0.59	1.00	(0.99-1.01)	0.90	1.01	(1.01-1.01)	<0.01			
**GEE**	**COVID-19**	0.87	(0.74-1.02)	0.09	0.89	(0.85-0.92)	<0.01	0.96	(0.95-0.98)	<0.01			
	**Time**	1.00	(0.99-1.01)	0.83	1.01	(1.00-1.01)	<0.01	0.97	(0.97-0.97)	<0.01			
	**Trend**	1.00	(0.99-1.02)	0.70	1.00	(1.00-1.01)	0.83	1.01	(1.01-1.01)	<0.01			

For peer review only

STROBE Statement—checklist of items that should be included in reports of observational studies

	Item No.	Recommendation	Page No.	Relevant text from manuscript
**Title and abstract**	1	(*a*) Indicate the study's design with a commonly used term in the title or the abstract	***1***	Title and abstract
		(*b*) Provide in the abstract an informative and balanced summary of what was done and what was found	***2***	Methods and Results section of abstract
**Introduction**				
Background/rationale	2	Explain the scientific background and rationale for the investigation being reported	3	Introduction section
Objectives	3	State specific objectives, including any prespecified hypotheses	4	Last paragraph of Introduction section
**Methods**				
Study design	4	Present key elements of study design early in the paper	4,5	Timeline of events and data sub-sections in the methods section
Setting	5	Describe the setting, locations, and relevant dates, including periods of recruitment, exposure, follow-up, and data collection	8	Data subsection in Methods section. Data obtained from National Health Information System.
Participants	6	(*a*) *Cohort study*—Give the eligibility criteria, and the sources and methods of selection of participants. Describe methods of follow-up<br>*Case-control study*—Give the eligibility criteria, and the sources and methods of case ascertainment and control selection. Give the rationale for the choice of cases and controls<br>*Cross-sectional study*—Give the eligibility criteria, and the sources and methods of selection of participants	8	All health facilities that report to the National Health Information database were included. Aggregated health facility data was extracted and therefore no individual data was collected.
		(*b*) *Cohort study*—For matched studies, give matching criteria and number of exposed and unexposed<br>*Case-control study*—For matched studies, give matching criteria and the number of controls per case		
Variables	7	Clearly define all outcomes, exposures, predictors, potential confounders, and effect modifiers. Give diagnostic criteria, if applicable	10	Segmented regression subsection

For peer review only - http://bmjopen.bmj.com/site/about/guidelines.xhtml

For peer review only

Data sources/ measurement	8*	For each variable of interest, give sources of data and details of methods of assessment (measurement). Describe comparability of assessment methods if there is more than one group	*8*	*Extracted data sub-section*
Bias	9	Describe any efforts to address potential sources of bias	8,9	Missing data handling
Study size	10	Explain how the study size was arrived at	8	Data sources subsection-All health facilities reporting in the national health information database

Continued on next page

For peer review only

Quantitative variables	11	Explain how quantitative variables were handled in the analyses. If applicable, describe which groupings were chosen and why	8-9	Statistical analysis subsection
Statistical methods	12	(*a*) Describe all statistical methods, including those used to control for confounding	8-11	Statistical analysis subsection
		(*b*) Describe any methods used to examine subgroups and interactions	NA	No subgroup analysis was carried out
		(*c*) Explain how missing data were addressed	9	Handling missing data sub-section
		(*d*) *Cohort study*—If applicable, explain how loss to follow-up was addressed<br>*Case-control study*—If applicable, explain how matching of cases and controls was addressed<br>*Cross-sectional study*—If applicable, describe analytical methods taking account of sampling strategy	NA	No loss to follow up in the study period.
		(*e*) Describe any sensitivity analyses	11	Last paragraph of Methods section
**Results**				
Participants	13*	(a) Report numbers of individuals at each stage of study—eg numbers potentially eligible, examined for eligibility, confirmed eligible, included in the study, completing follow-up, and analysed	9	Number of health facilities analysed provided in the last sentence of subsection on Handling missing data
		(b) Give reasons for non-participation at each stage	NA	No individual data
		(c) Consider use of a flow diagram	NA	
Descriptive data	14*	(a) Give characteristics of study participants (eg demographic, clinical, social) and information on exposures and potential confounders	NA	No individual patient's data
		(b) Indicate number of participants with missing data for each variable of interest	9	Missing data across hospitals provided
		(c) *Cohort study*—Summarise follow-up time (eg, average and total amount)	NA	
Outcome data	15*	*Cohort study*—Report numbers of outcome events or summary measures over time	*12*	Statistical Process Control (SPC) charts showing changes of monthly attendance post-COVID19 using the standard deviation from mean of baseline period (pre-COVID19) Figure 3
		Case-control study—Report numbers in each exposure category, or summary measures of exposure		
		Cross-sectional study—Report numbers of outcome events or summary measures		

For peer review only - http://bmjopen.bmj.com/site/about/guidelines.xhtml

Main results	16	(*a*) Give unadjusted estimates and, if applicable, confounder-adjusted estimates and their precision (eg, 95% confidence interval). Make clear which confounders were adjusted for and why they were included	12	Table 2
		(*b*) Report category boundaries when continuous variables were categorized	NA	No continuous variables were categorized
		(*c*) If relevant, consider translating estimates of relative risk into absolute risk for a meaningful time period	13	Results were rate ratios, translated to percentages to quantify the changes

Continued on next page

For peer review only

Other analyses	17	Report other analyses done—eg analyses of subgroups and interactions, and sensitivity analyses	13	Sensitivity analyses section
**Discussion**				
Key results	18	Summarise key results with reference to study objectives	14	Discussion section
Limitations	19	Discuss limitations of the study, taking into account sources of potential bias or imprecision. Discuss both direction and magnitude of any potential bias	17	Limitations subsection
Interpretation	20	Give a cautious overall interpretation of results considering objectives, limitations, multiplicity of analyses, results from similar studies, and other relevant evidence	16	Strengths and implications of study subsection
Generalisability	21	Discuss the generalisability (external validity) of the study results	16,17	Strengths and implications of study subsection and Conclusion subsection
**Other information**				
Funding	22	Give the source of funding and the role of the funders for the present study and, if applicable, for the original study on which the present article is based	18	Funding subsection

*Give information separately for cases and controls in case-control studies and, if applicable, for exposed and unexposed groups in cohort and cross-sectional studies.

Note: An Explanation and Elaboration article discusses each checklist item and gives methodological background and published examples of transparent reporting. The STROBE checklist is best used in conjunction with this article (freely available on the Web sites of PLoS Medicine at http://www.plosmedicine.org/, Annals of Internal Medicine at http://www.annals.org/, and Epidemiology at http://www.epidem.com/). Information on the STROBE Initiative is available at www.strobe-statement.org.

For peer review only
